# Supplementary material for: Control of water for high-yield and low-cost sustainable electrochemical synthesis of uniform monolayer graphene oxide
Source: Nat Commun. 2025 Jan 16;16:727. doi: 10.1038/s41467-025-56121-4 (PMC11739590; doi:10.1038/s41467-025-56121-4)
Supplement: Supplementary file 1 — Supplementary Information [file 41467_2025_56121_MOESM1_ESM.pdf]

Supplementary Materials for

Control of water for high-yield and low-cost sustainable electrochemical synthesis of uniform monolayer graphene oxide

Jiaqi Guo[1,2,4], Songfeng Pei[1,2,4]*, Kun Huang[1,2], Qing Zhang[1,2], Xizhong Zhou[1,2], Jinmeng Tong[1,2], Zhibo Liu[1,2], Hui-Ming Cheng[1,2,3], Wencai Ren[1,2]*

[1]Shenyang National Laboratory for Materials Science, Institute of Metal Research, Chinese Academy of Sciences, 72 Wenhua Road, Shenyang 110016, P. R. China.

[2]School of Materials Science and Engineering, University of Science and Technology of China, 72 Wenhua Road, Shenyang 110016, P. R. China.

[3]Chinese Academy of Sciences Shenzhen Institutes of Advanced Technology, Institute of Technology for Carbon Neutrality, No. 1068, Xueyuan Road, Nanshan District, Shenzhen, Guangdong Province, Shenzhen, CHINA

[4]These authors contributed equally: Jiaqi Guo, Songfeng Pei

*Corresponding author. Email: sfpei@imr.ac.cn and wcren@imr.ac.cn

Table of Contents

Supplementary Figures……………………………………………………2

Supplementary Tables……………………………………………………36

Supplementary Notes……………………………………………………..44

Supplementary References……………………………………………68

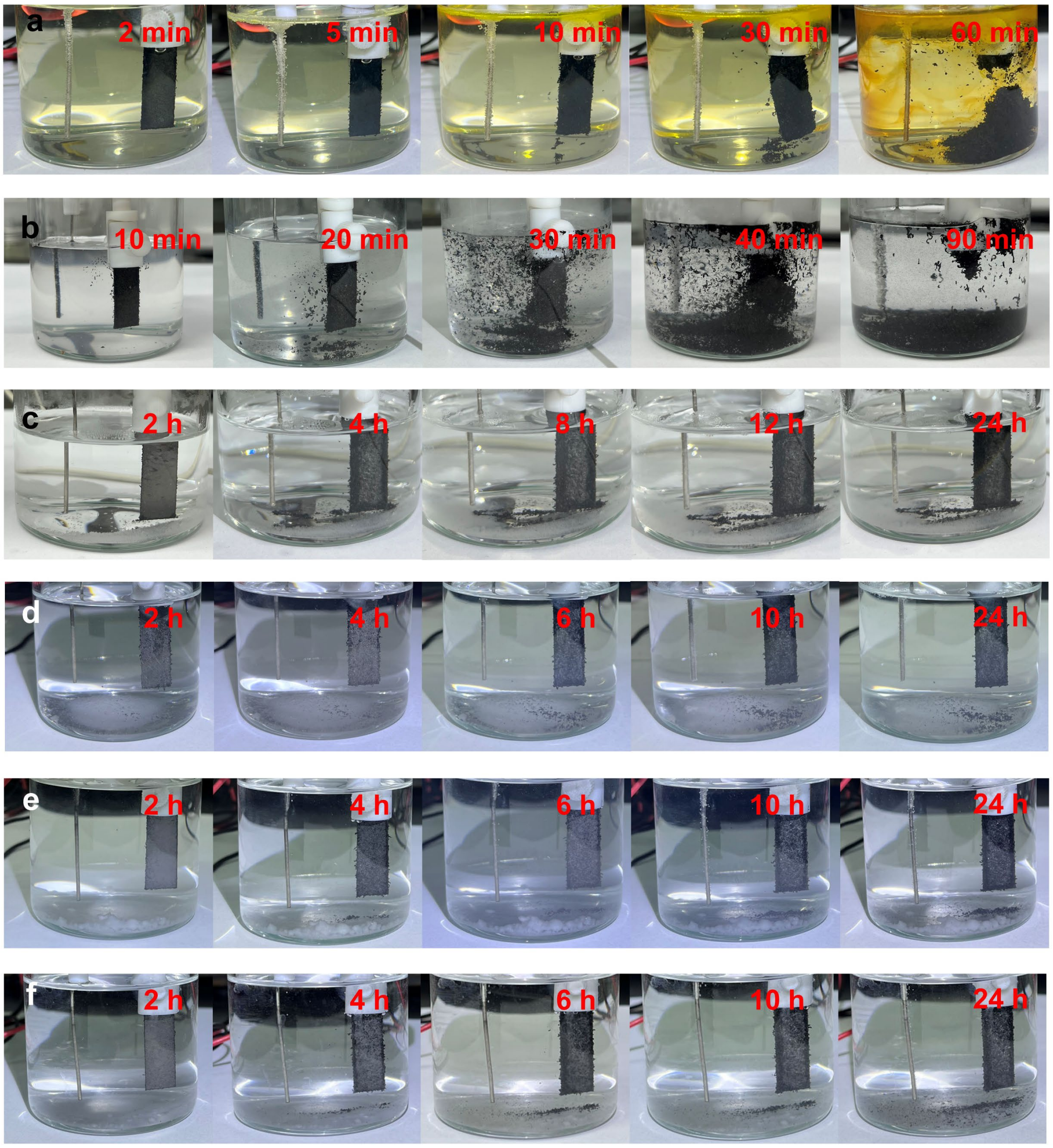

Supplementary Figure 1. Morphology changes of FGP anodes during EC reaction in non-aqueous electrolytes. a, [BMIm][BF_4]. **b,** $LiClO_4$(0.5 mol L^{-1}) + polycarbonate. **c,** Methanol + KPF_6(KPF_6 saturated). **d,** Ethanol + KPF_6(KPF_6 saturated). **e,** Methanol + $LiPF_6$($LiPF_6$ saturated). f, Ethanol+ $LiPF_6$($LiPF_6$ saturated).

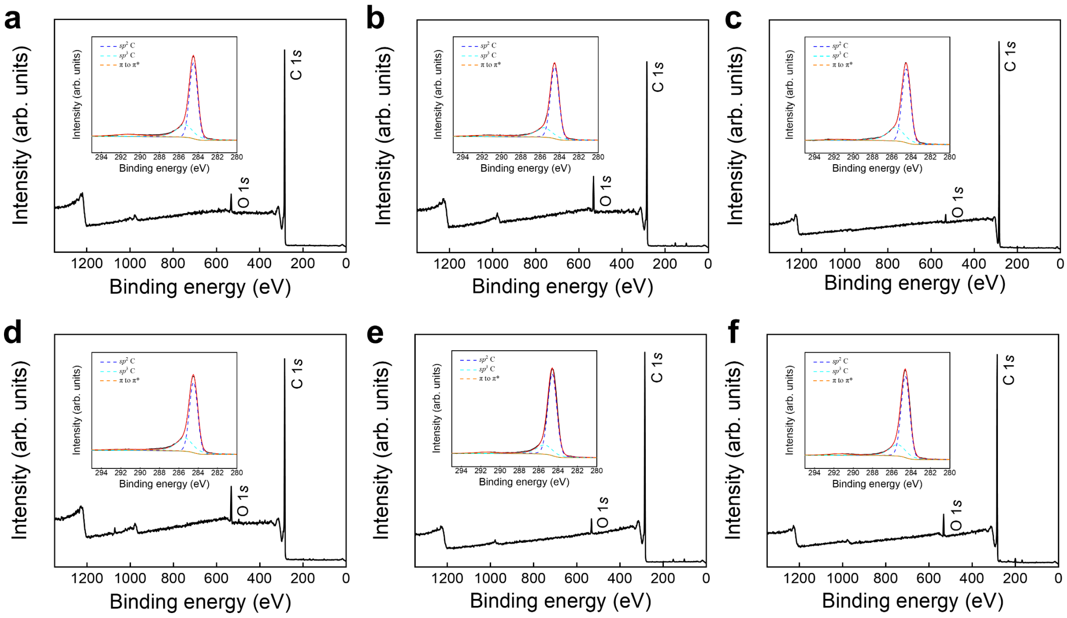

Supplementary Figure 2. XPS survey spectra and C 1*s* spectra (inset) of the products synthesized by EC reaction of FGP in non-aqueous electrolytes. a, [BMIm][BF_4]. **b,** $LiClO_4$ (0.5 mol L^{-1}) + polycarbonate. **c,** Methanol + KPF_6 (KPF_6 saturated). **d,** Ethanol + KPF_6 (KPF_6 saturated). **e,** Methanol + $LiPF_6$ ($LiPF_6$ saturated). **f,** Ethanol + $LiPF_6$ ($LiPF_6$ saturated).

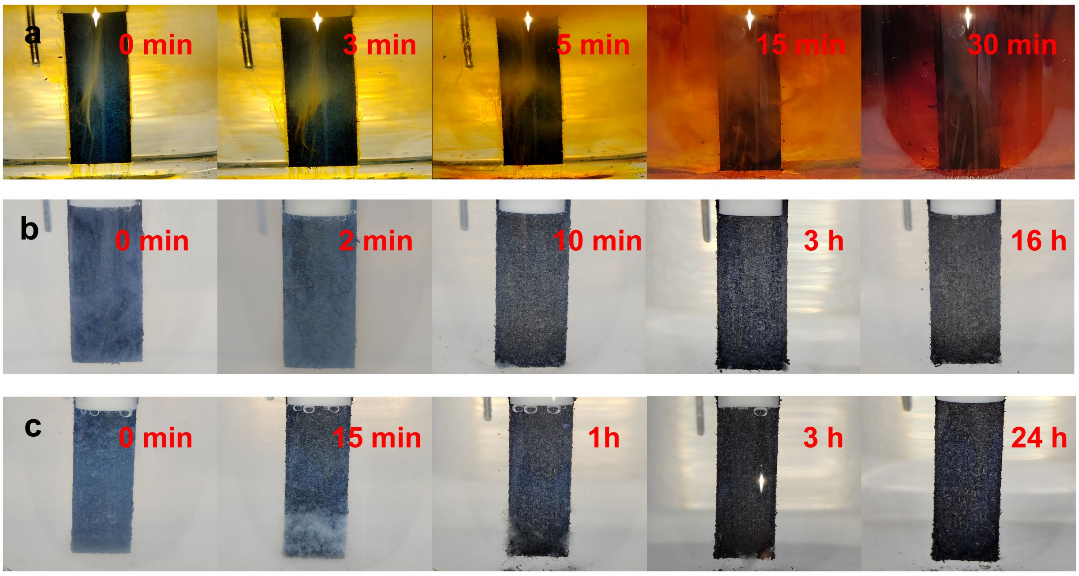

Supplementary Figure 3. Morphology changes of SA-GIC-I anodes during EC reaction in non-aqueous electrolytes. a, [BMIm][BF_4]. **b,** Ethanol + $NaPF_6$($NaPF_6$ saturated).. **c,** Ethanol + KPF_6(KPF_6 saturated).

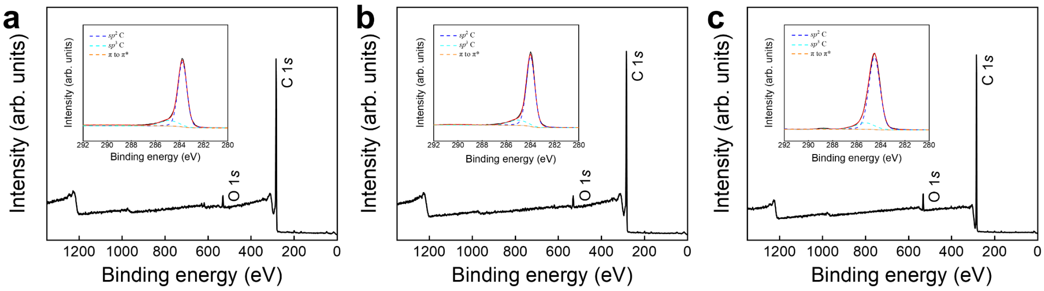

Supplementary Figure 4. XPS survey spectra and C 1*s* spectra (inset) of the products synthesized by EC reaction of SA-GIC-I in non-aqueous electrolytes. a, [BMIm][BF_4]. **b,** Ethanol + $NaPF_6$ ($NaPF_6$ saturated). **c,** Ethanol + KPF_6 (KPF_6 saturated).

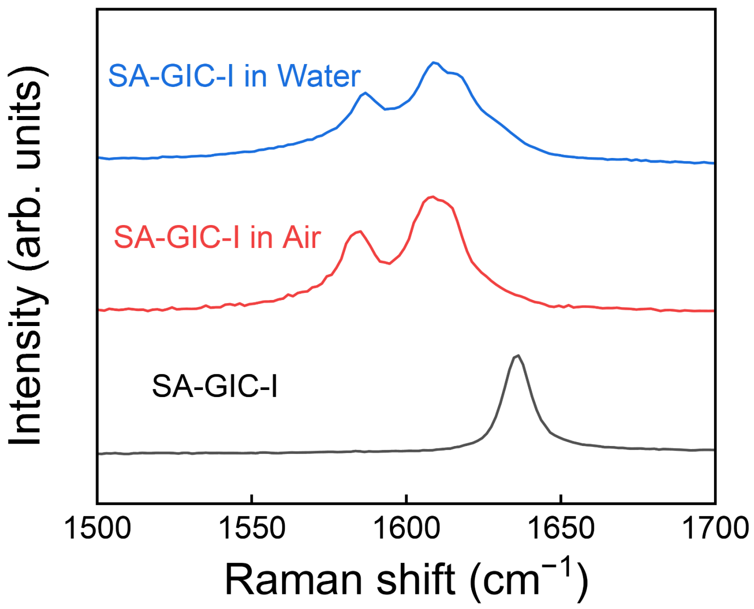

Supplementary Figure 5. Raman spectra of freshly synthesized SA-GIC-I samples and deintercalated SA-GIC-I samples after standing in humid air for 50 mins (SA-GIC-I in Air) and dipping in water for 5 s (SA-GIC-I in water).

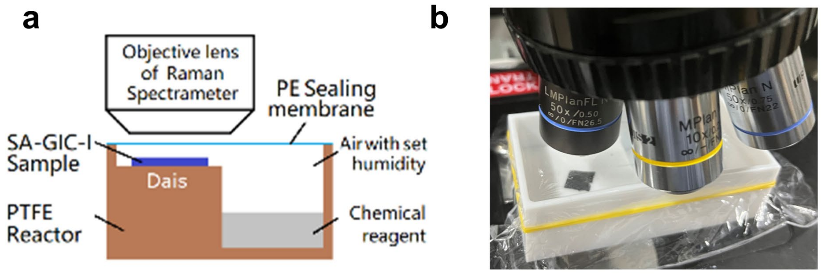

Supplementary Figure 6. Device for in-situ Raman spectroscopic investigation. a, schematic diagram of the cross-sectional structure of the device. **b,** Photo of the device.

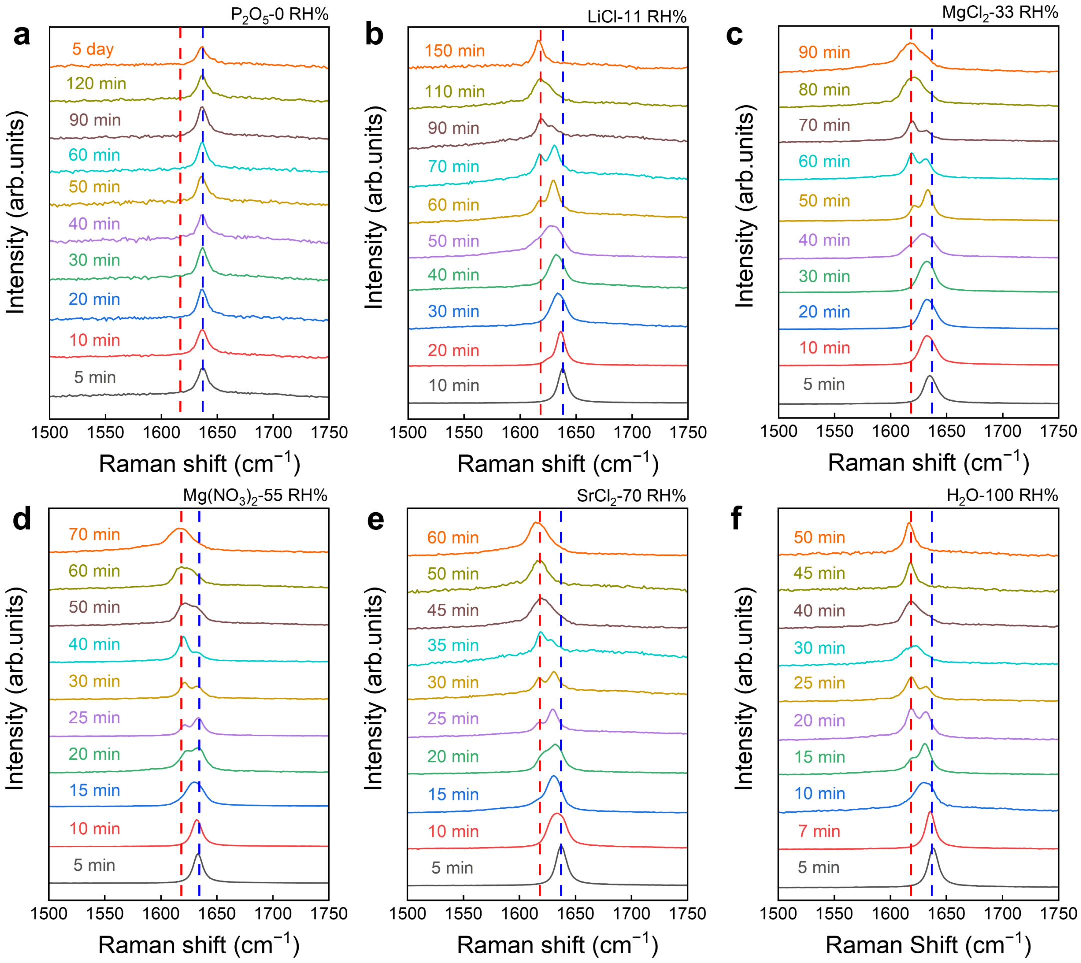

Supplementary Figure 7. The evolution of Raman spectra of SA-GIC-I samples in air with different humidity. a, 0% RH. **b,** 11% RH. **c,** 33% RH. **d,** 55% RH. **e,** 70% RH. **f,** 100% RH.

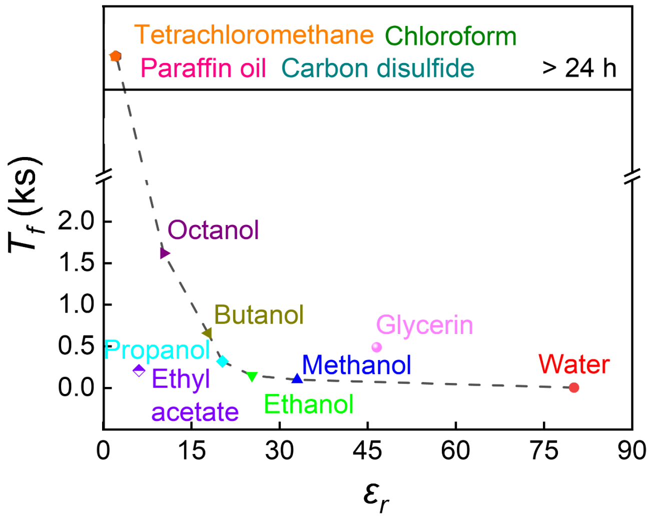

Supplementary Figure 8. The relationship between the time needed for the fully fading of blue color of the SA-GIC-I surface (T_f) and relative dielectric constant (ε_r) of solvents.

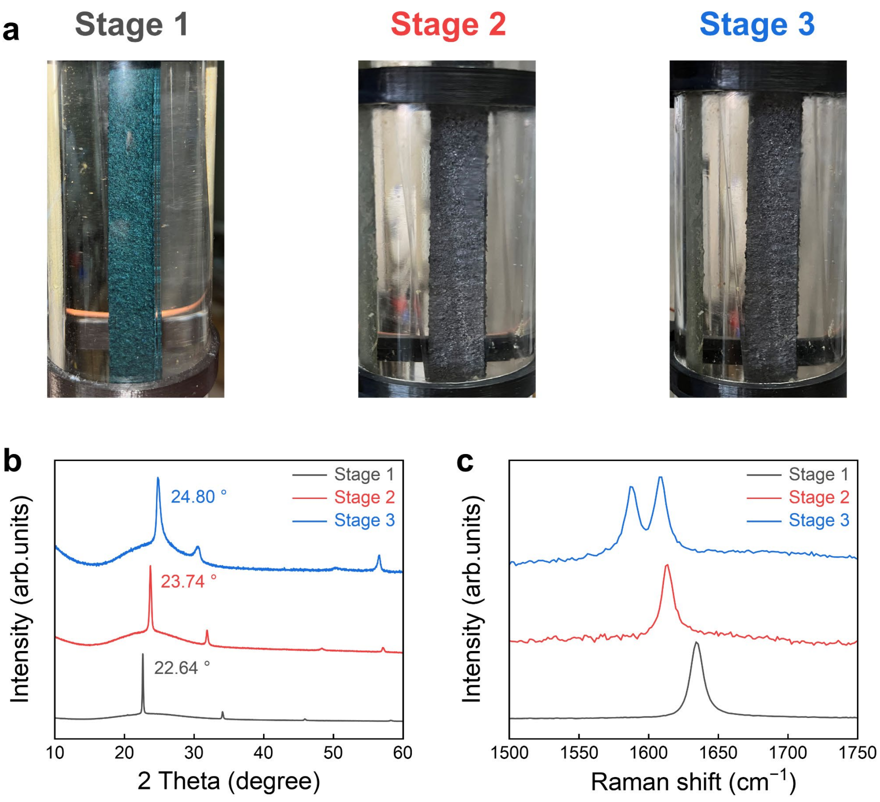

Supplementary Figure 9. Characterization of intercalation products obtained at different voltages. a, Optical photograph. **b,** XRD patterns. **c,** Raman spectra.

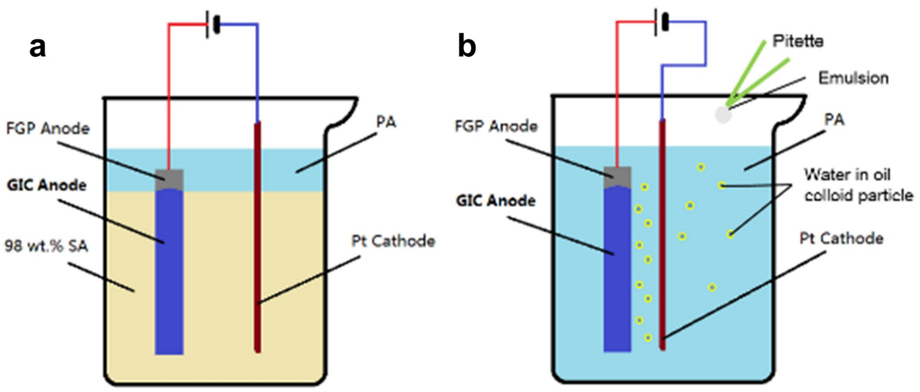

Supplementary Figure 10. Schematic diagrams of the device for emulsion electrolysis. a, Step I: EC intercalation. **b,** Step II: emulsion electrolysis.

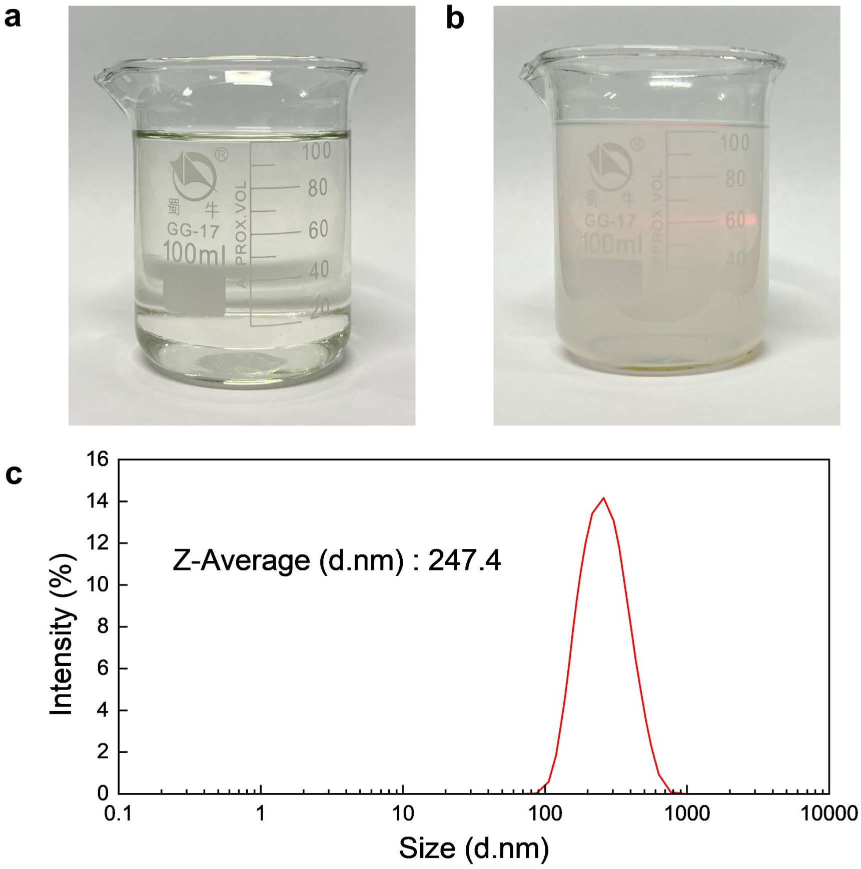

Supplementary Figure 11. Preparation and characteristics of water-in-oil colloidal emulsion. a, Mixture of PA and water before high shear dispersion, where surfactant (Span-85) was pre-dissolved in PA. **b,** Colloidal emulsion obtained after shear dispersion. **c,** Particle size distribution of colloidal emulsion.

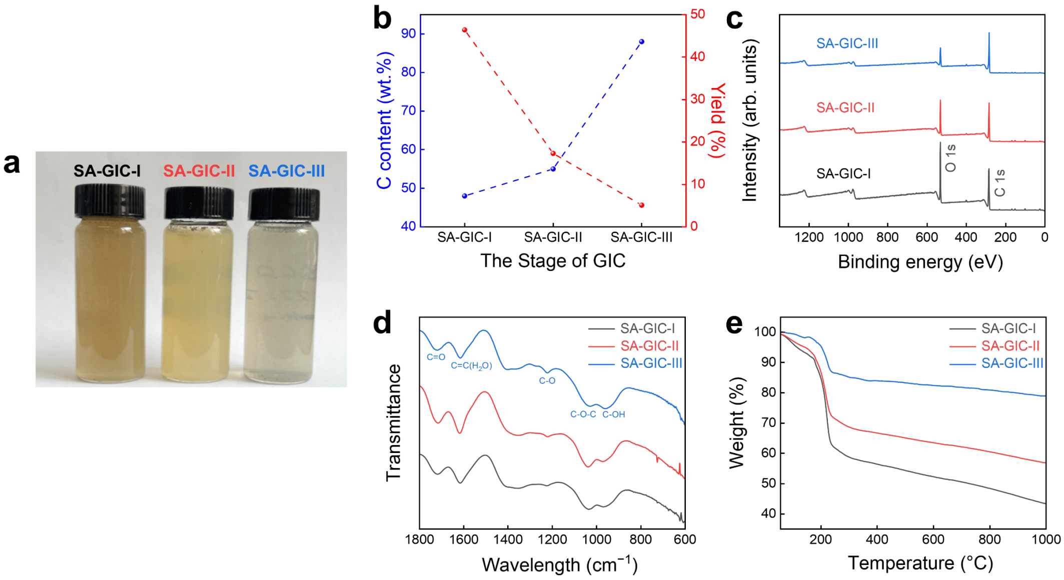

Supplementary Figure 12. Comparison of the morphologies and properties of emulsion electrolysis products synthesized from SA-GIC with different stages. a–b, photos of supernatants (**a**) and carbon content and yield of products (**b**). **c–f,** XPS spectra (**c**). FTIR spectra (**d**). and TG curves (**e**) of the products.

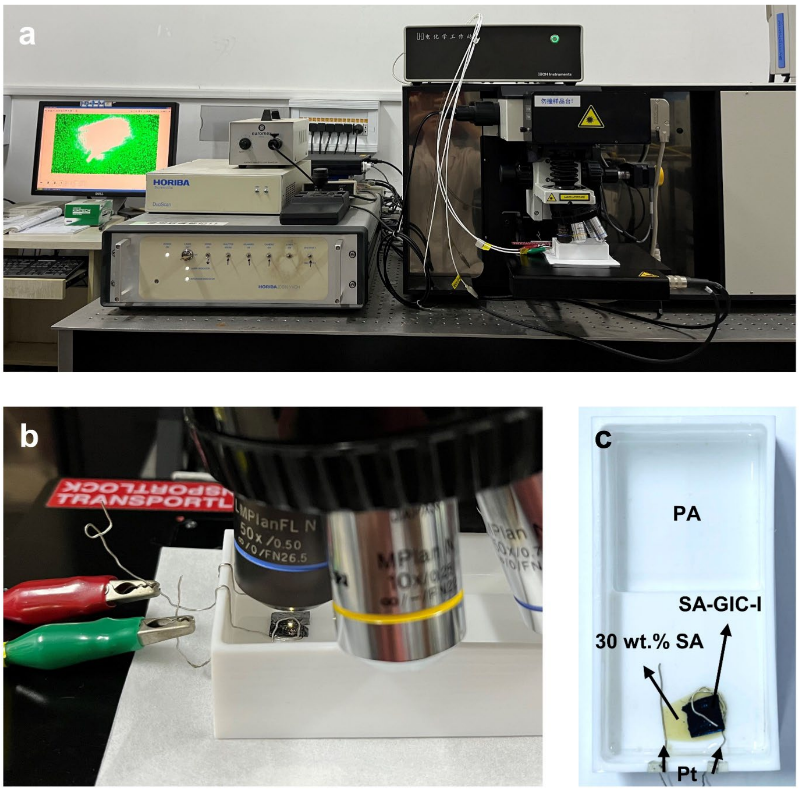

Supplementary Figure 13. Device for in-situ experimental investigations. a, The whole device for in-situ microscopic observation and Raman spectroscopic investigation. **b,** A homemade in-situ EC reactor. **c,** The EC reaction circuit in reactor using small electrolyte droplet to connect Pt cathode and SA-GIC-I anode.

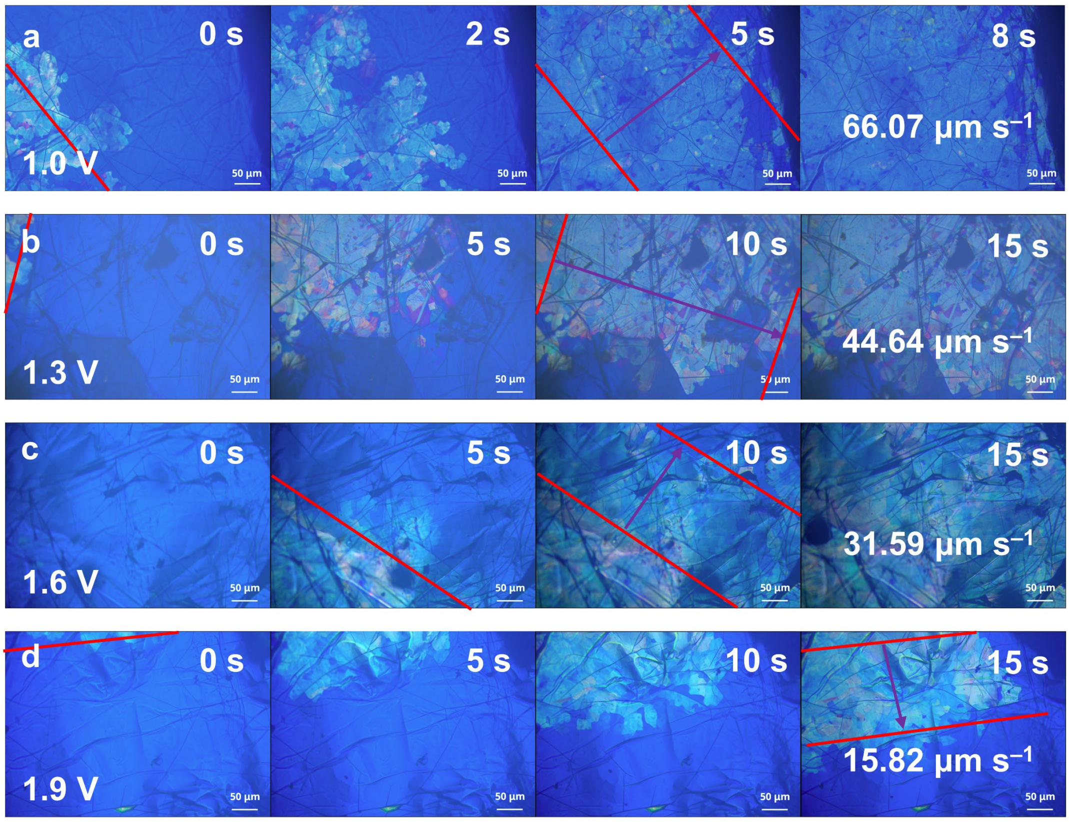

Supplementary Figure 14. The expanding of deintercalated areas on anode surface under different voltages in the range of 1.0 V~1.9 V.

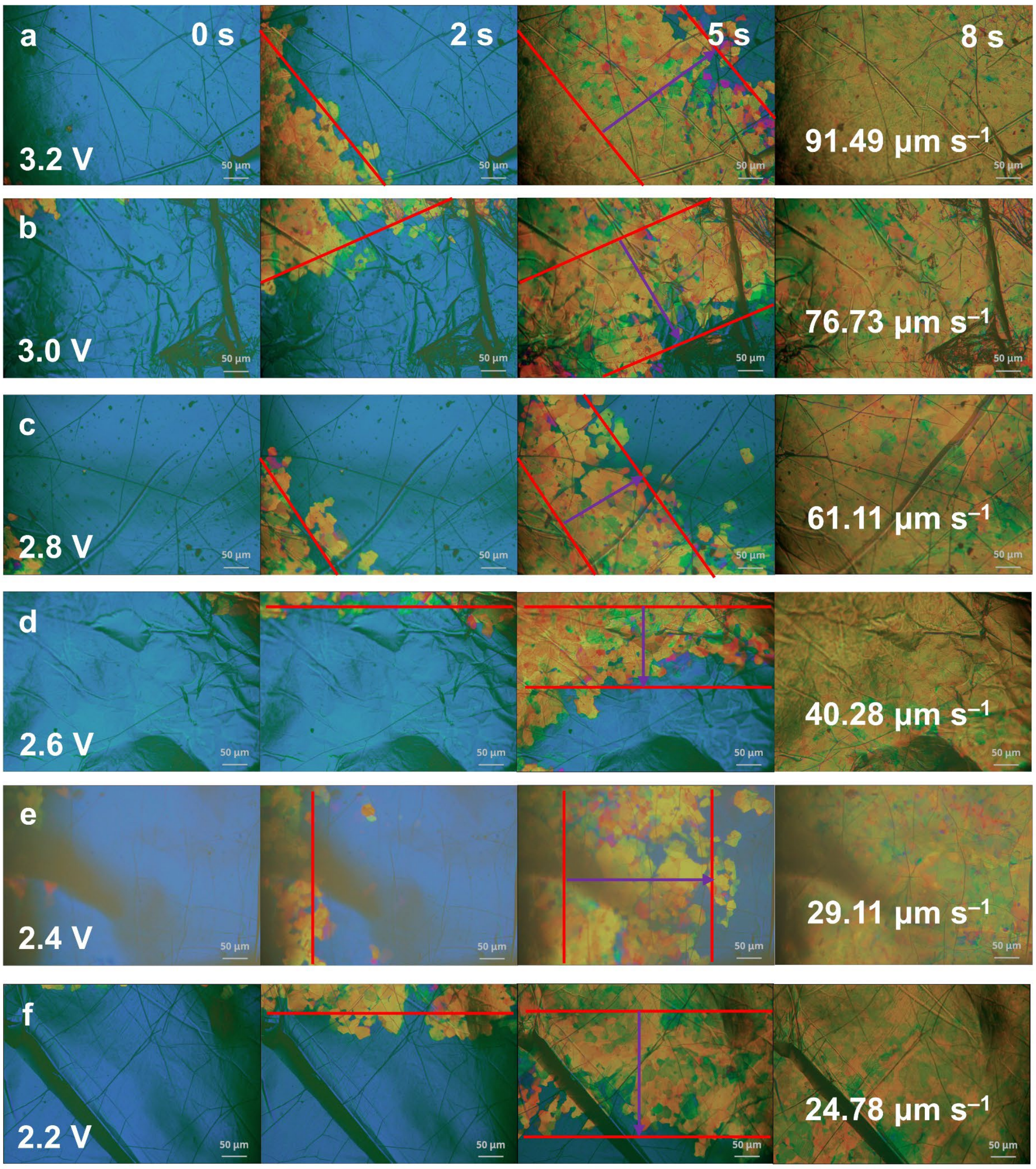

Supplementary Figure 15. The expanding of oxidized areas on anode surface under different voltages in the range of 2.2 V~3.2 V.

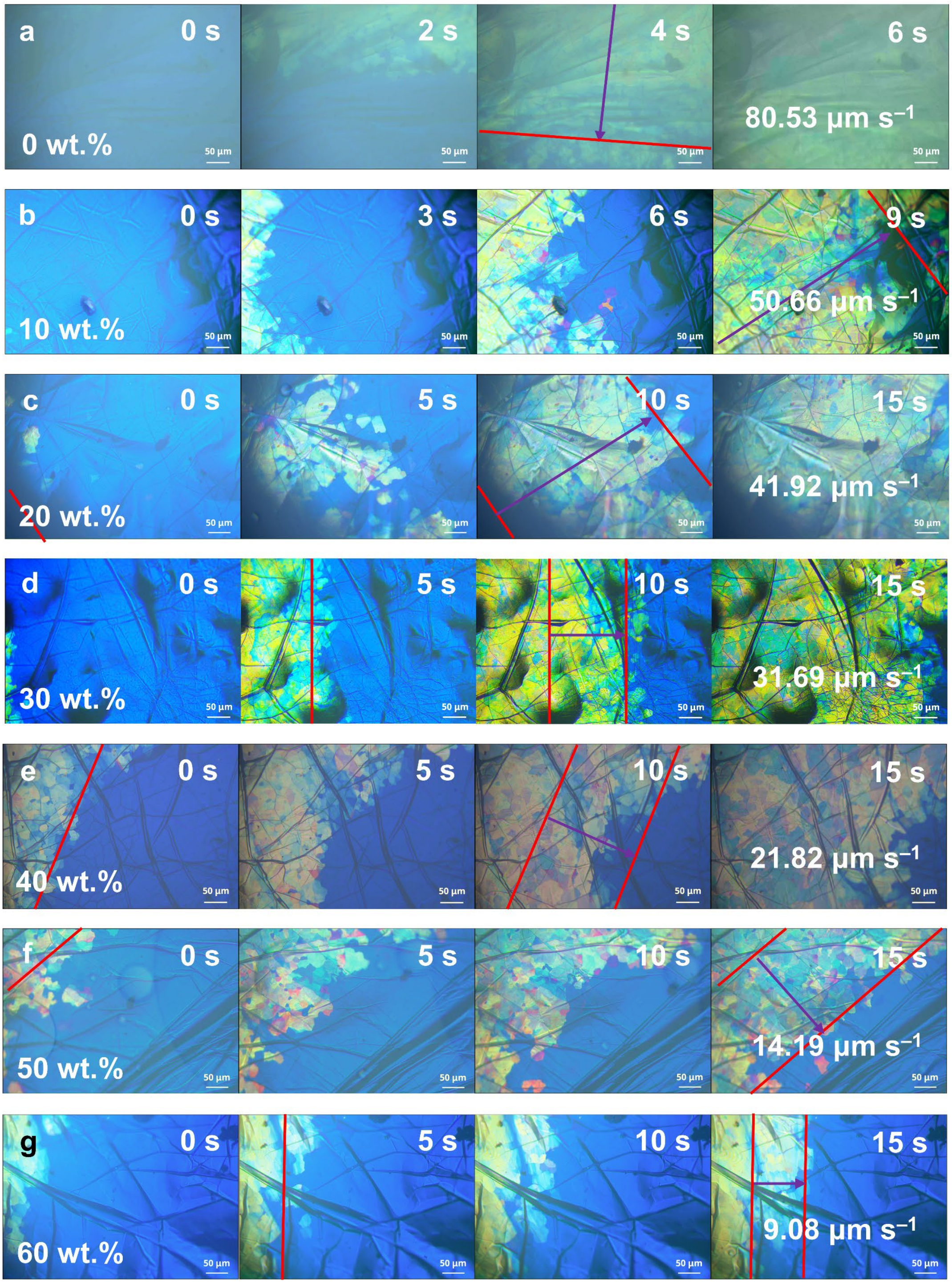

Supplementary Figure 16. The expanding of deintercalated or oxidized areas on anode surface with different SA electrolytes.

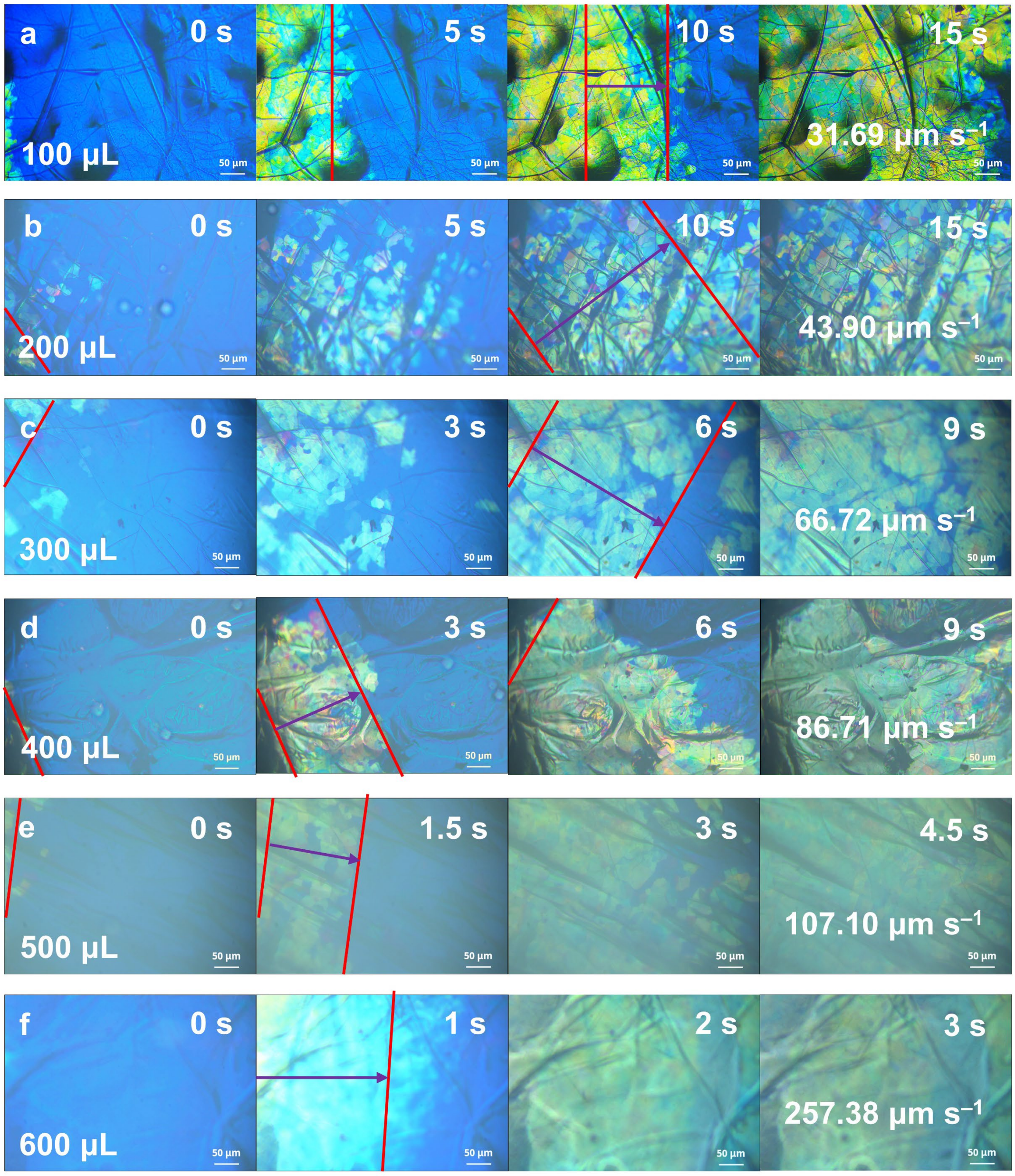

Supplementary Figure 17. The expanding of deintercalated or oxidized areas on anode surface with different SA electrolytes volume.

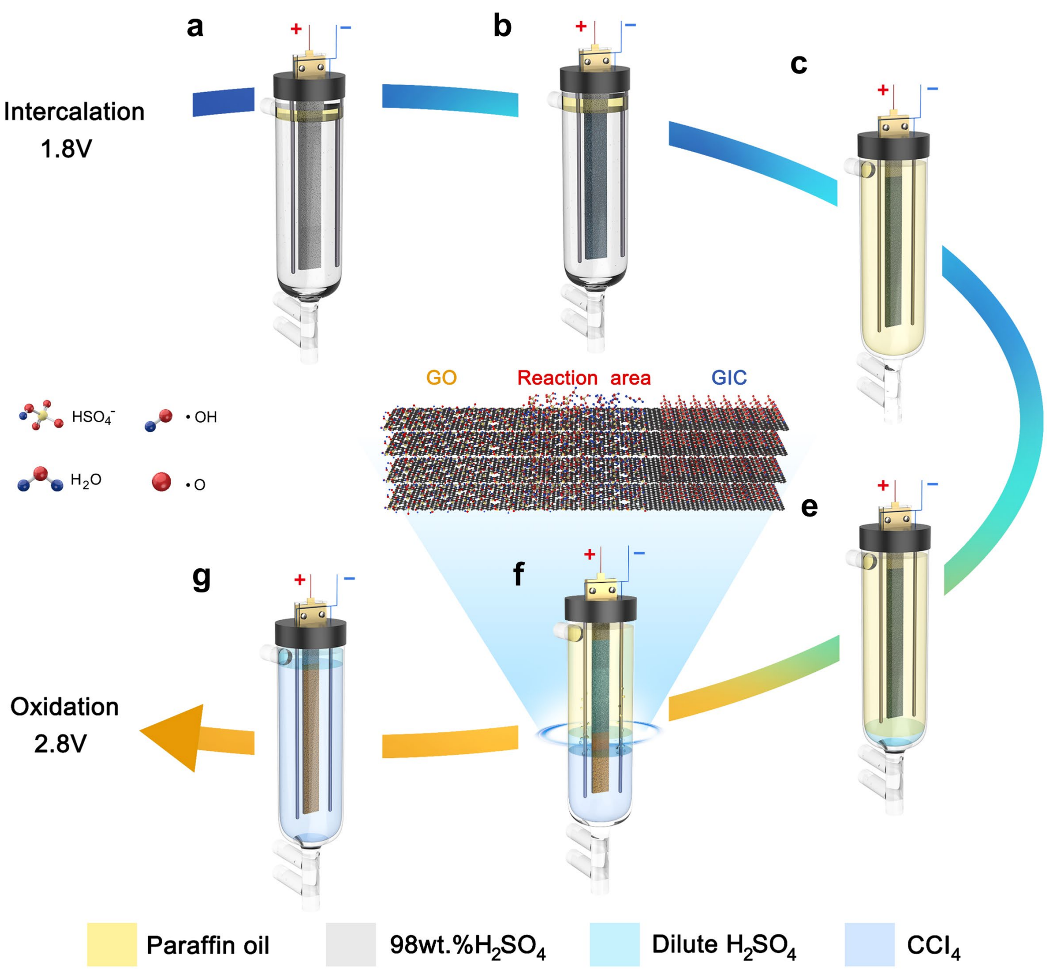

Supplementary Figure 18. Schematic of the basic process of LME.

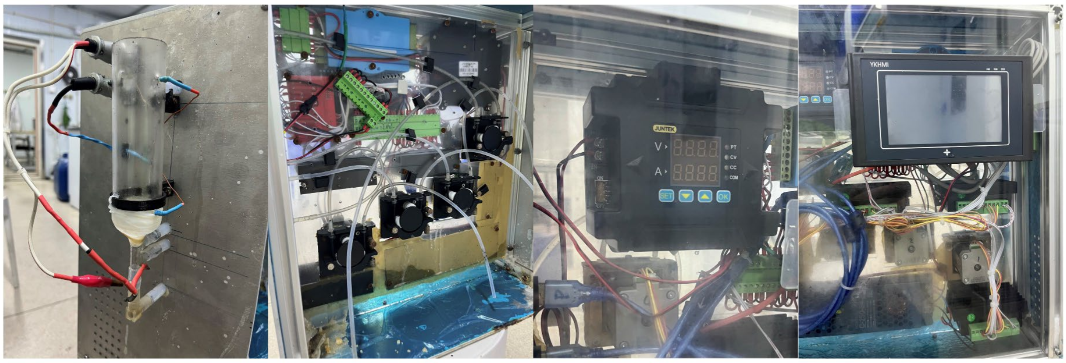

Supplementary Figure 19. The basic components of a homemade LME device. From left to right, reactor subsystem, fluid transport subsystem, electrical subsystem and automatic control subsystem.

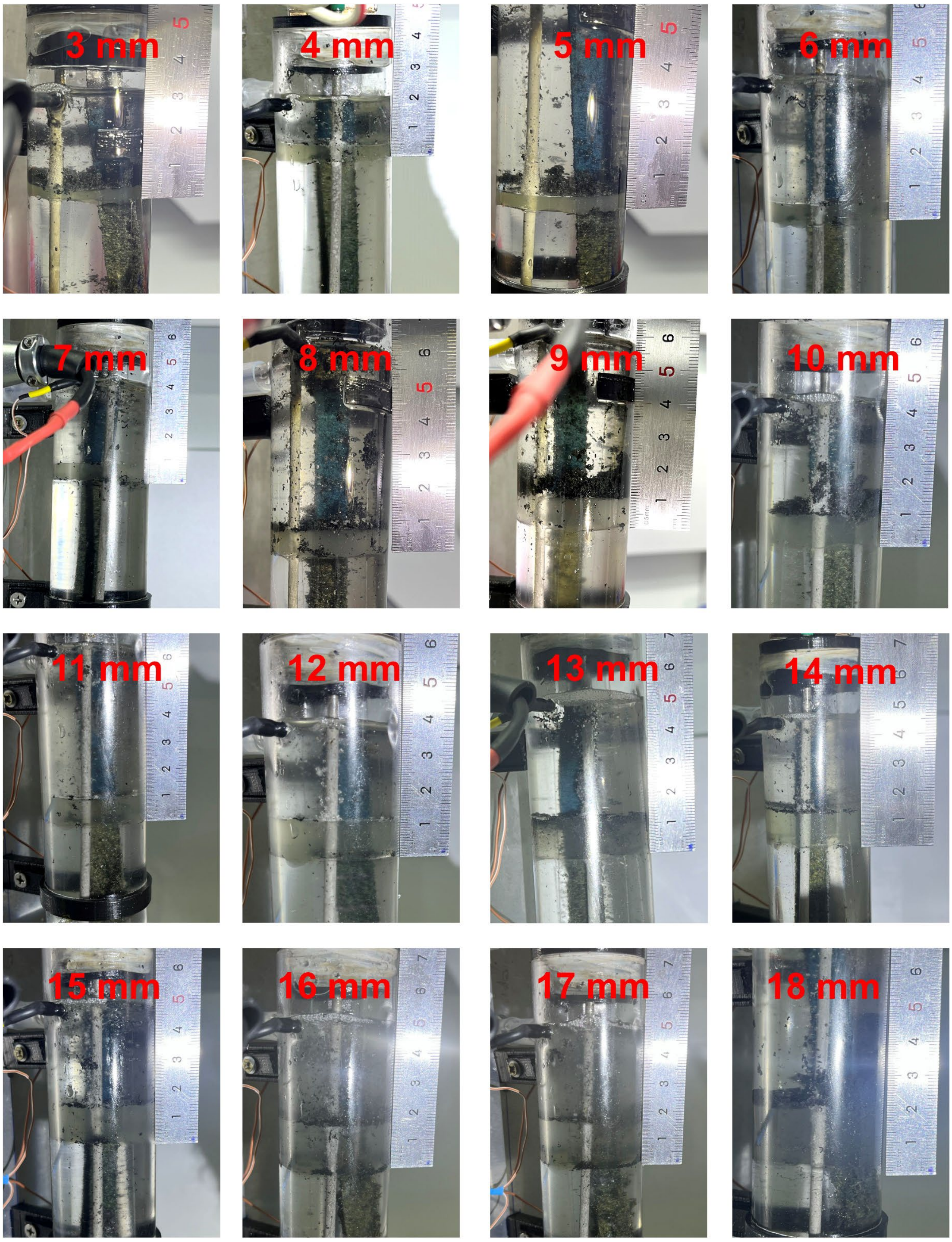

Supplementary Figure 20. LME oxidation processes with different LM thicknesses (3 mm to 18 mm) at a fixed LM rising speed (1.9 mm min^{-1}).

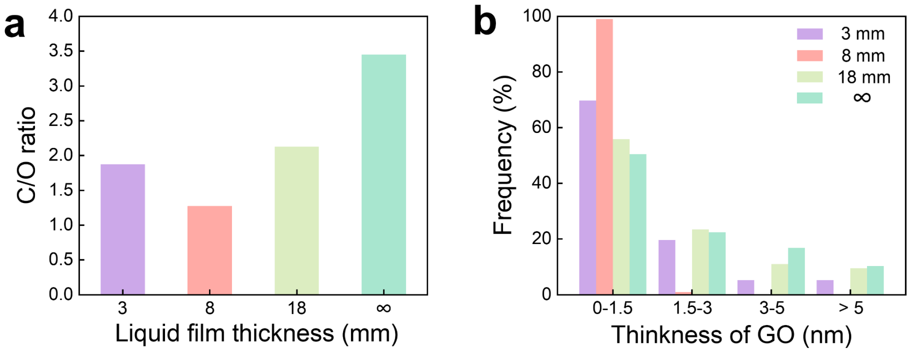

Supplementary Figure 21. (a) C/O ratio and (b) thickness distribution of the products synthesized with different LM thicknesses (3 mm, 8 mm, 18 mm, and ∞) at a fixed LM rising speed (1.9 mm min^{-1}).

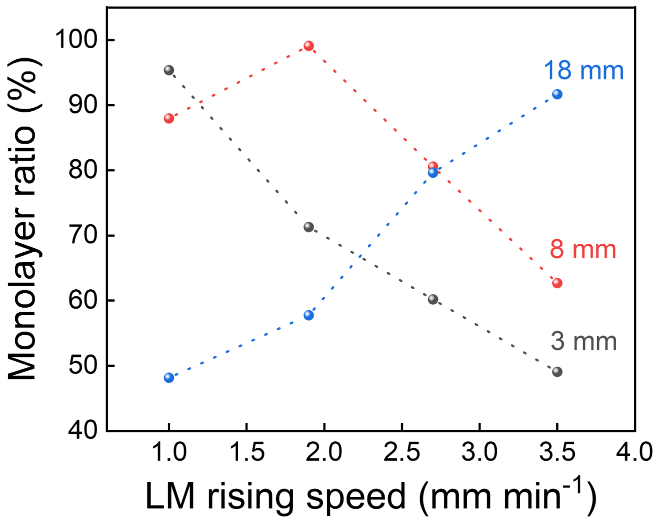

Supplementary Figure 22. Variation of monolayer ratio in the product with LM rising speed for different LM thicknesses.

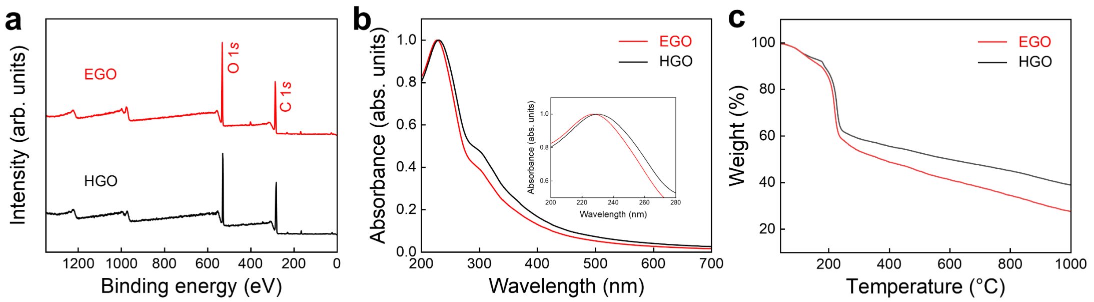

Supplementary Figure 23. Comparison of EGO and HGO products. a, XPS spectra. **b,** UV-Vis spectra. **c,** TG curves.

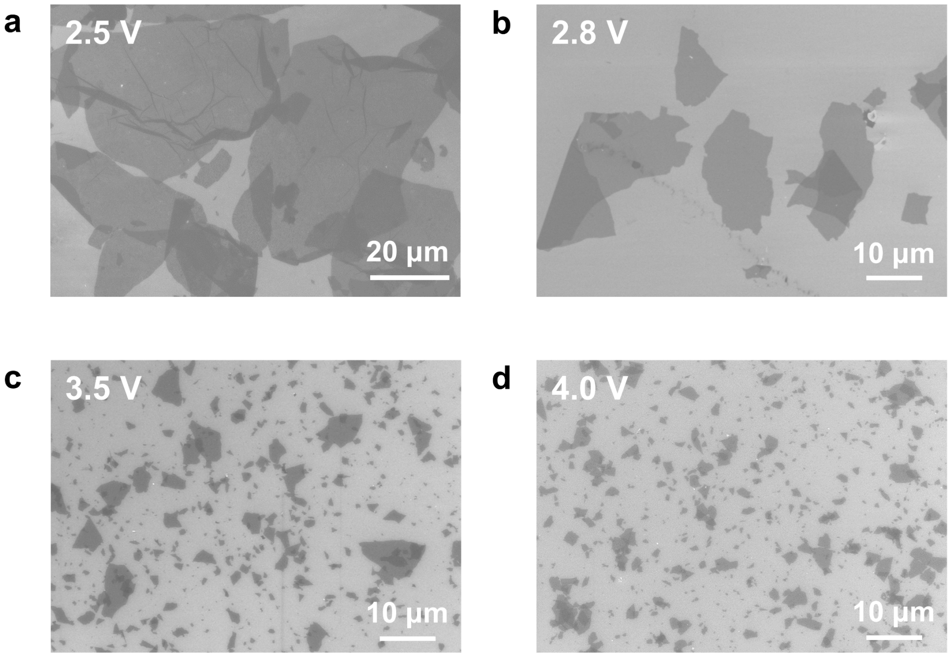

Supplementary Figure 24. Typical SEM images of GO flakes synthesized by LME at different voltages.

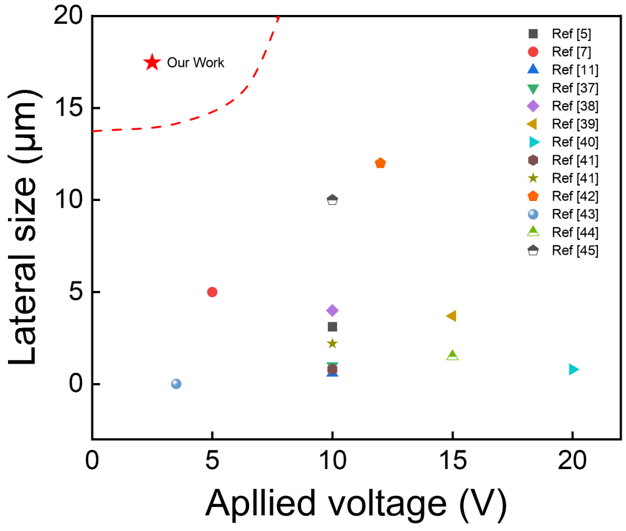

Supplementary Figure 25. The comparison of lateral size of EGO synthesized by different EC synthesis methods.

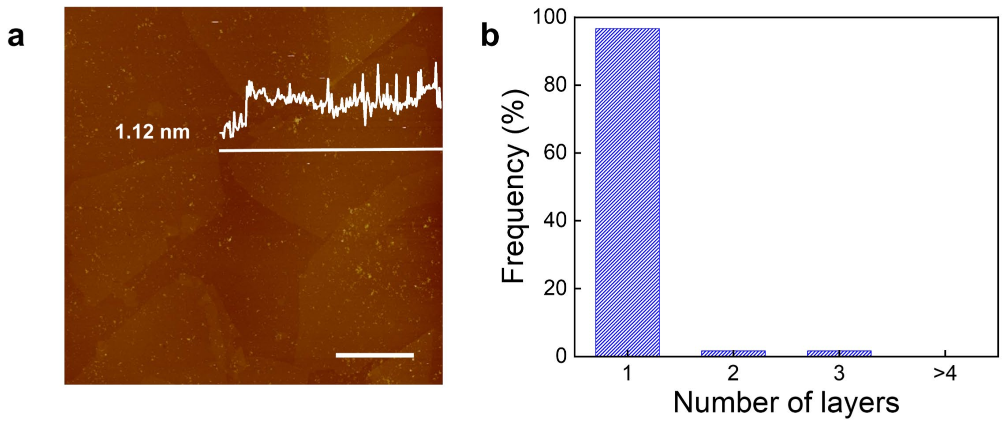

Supplementary Figure 26. Typical AFM image (a) and the number of layers distribution (b) of LEGO flakes synthesized by LME at 2.5 V. Scale bars: 10 μm.

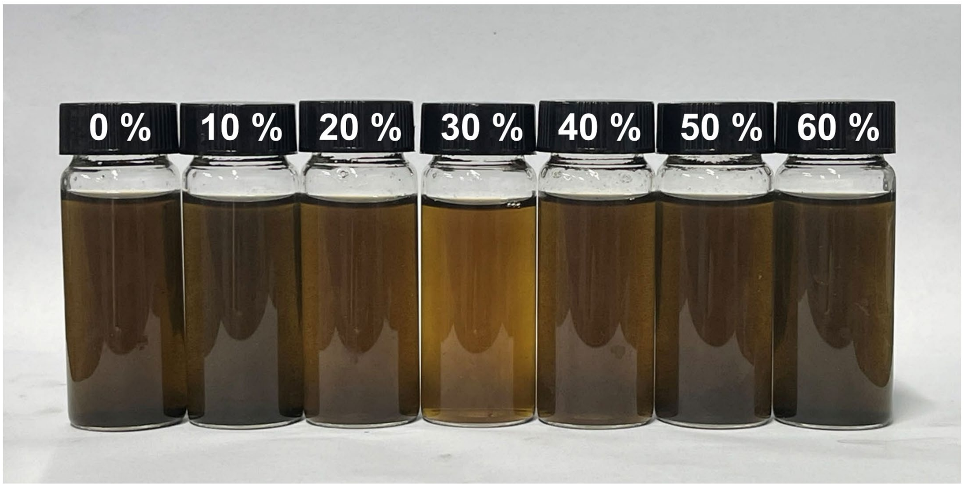

Supplementary Figure 27. Comparison of GO aqueous solutions synthesized by LME with different SA electrolytes.

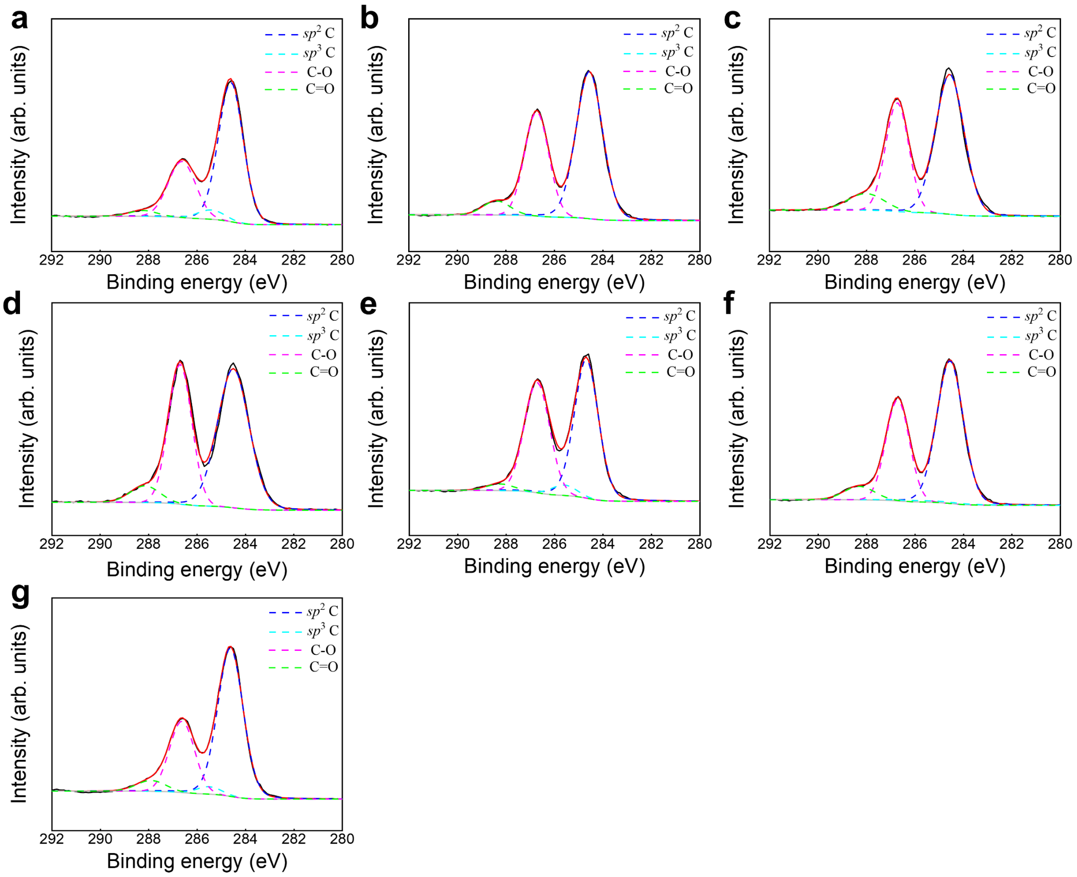

Supplementary Figure 28. XPS C1*s* fine spectra of GO products synthesized by LME with different SA electrolytes.

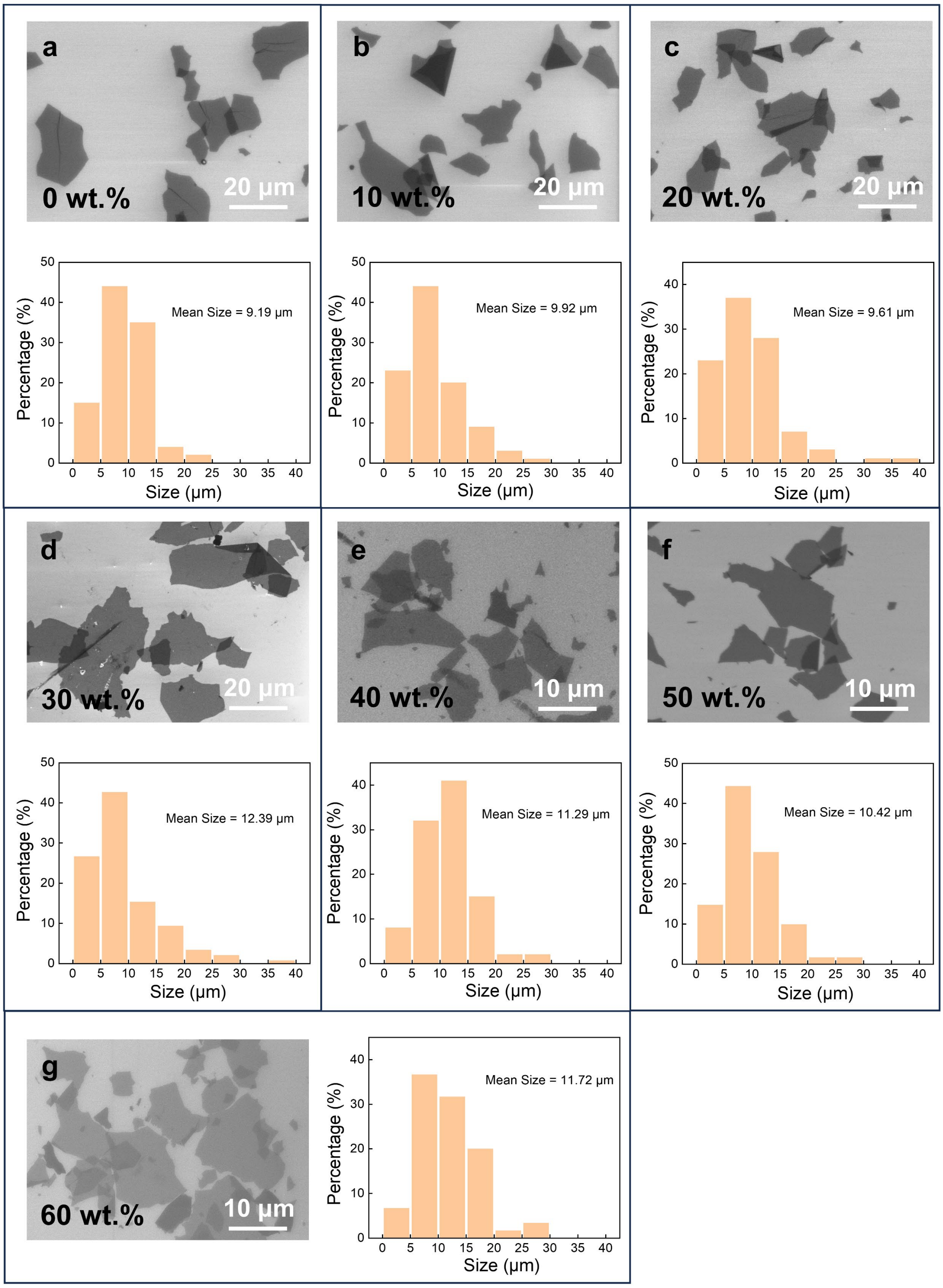

Supplementary Figure 29. SEM images and the corresponding lateral size distributions of GO products synthesized by LME with different SA electrolytes.

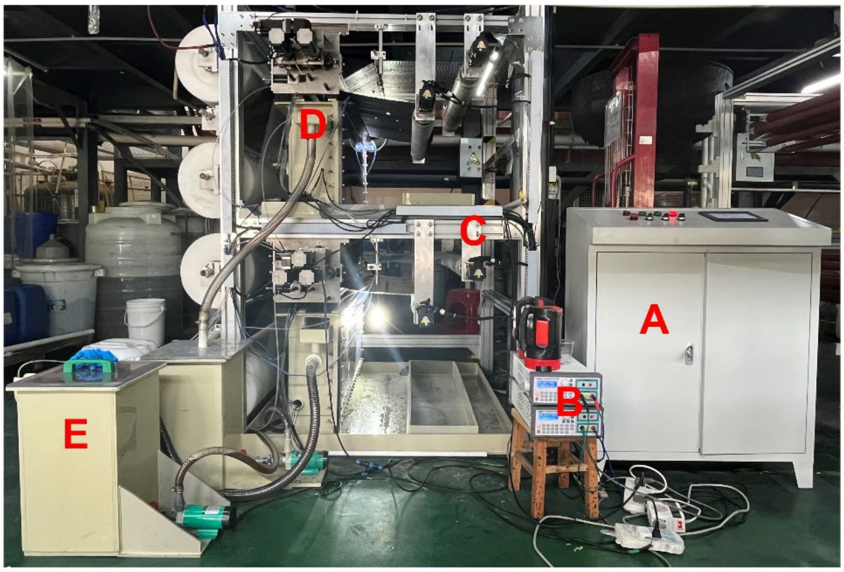

Supplementary Figure 30. Photo of industrial-scale LME equipment.

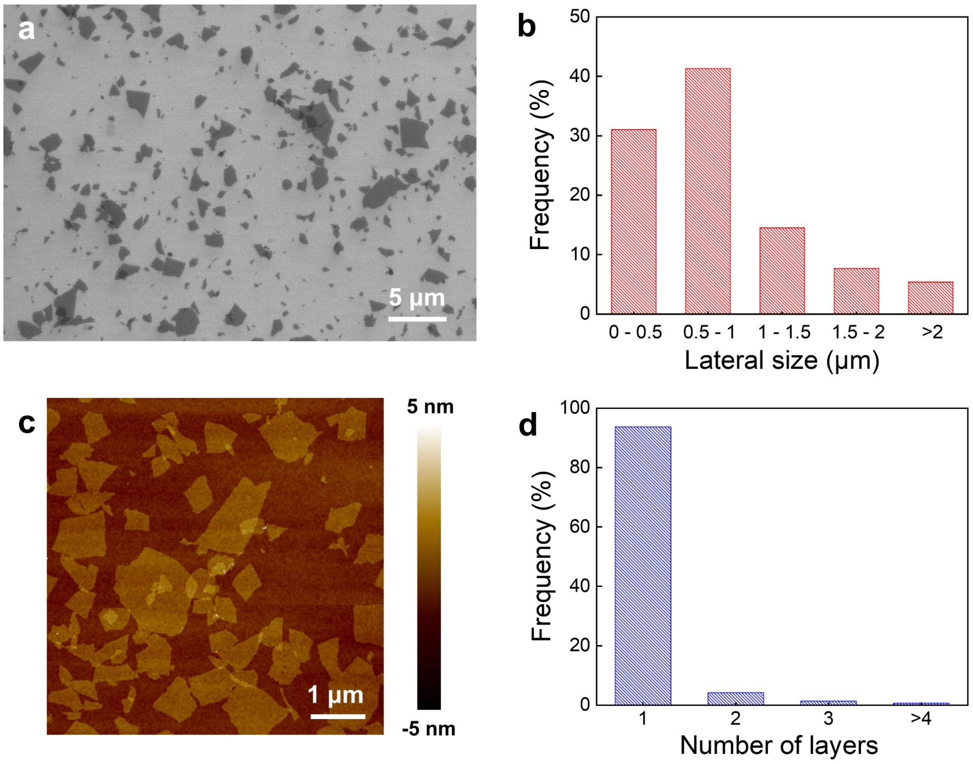

Supplementary Figure 31. Characterization of the lateral size and number of layers of EGO produced by industrial-scale LME.

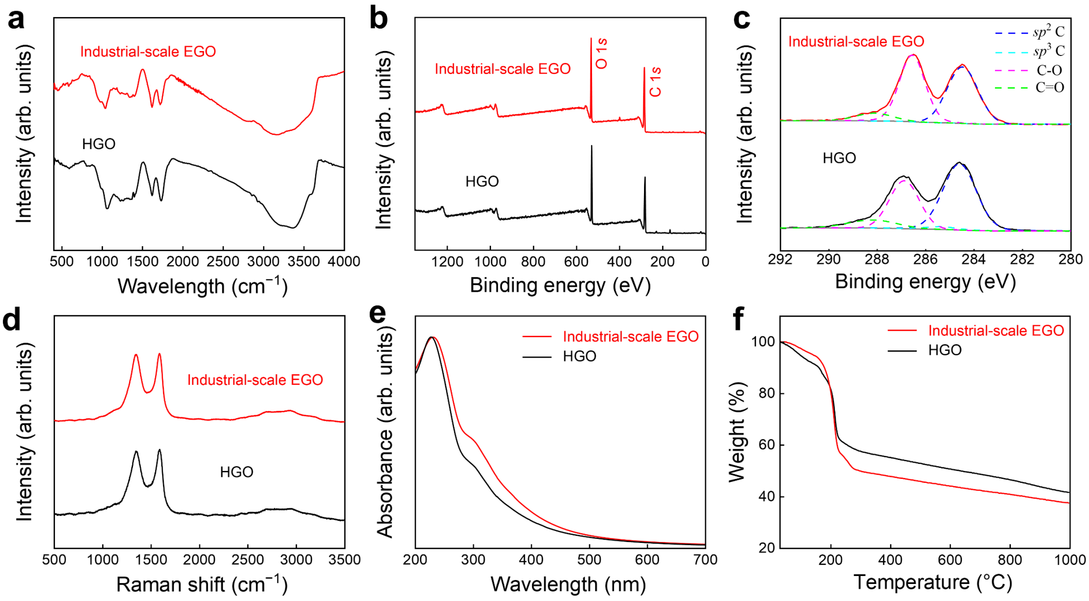

Supplementary Figure 32. Comparison of industrial-scale EGO produced by LME with lab-scale HGO. a, FTIR spectra. **b,** XPS survey spectra. **c,** XPS C1*s* spectra. **d,** Raman spectra. **e,** UV-Vis spectra. **f,** TG curves.

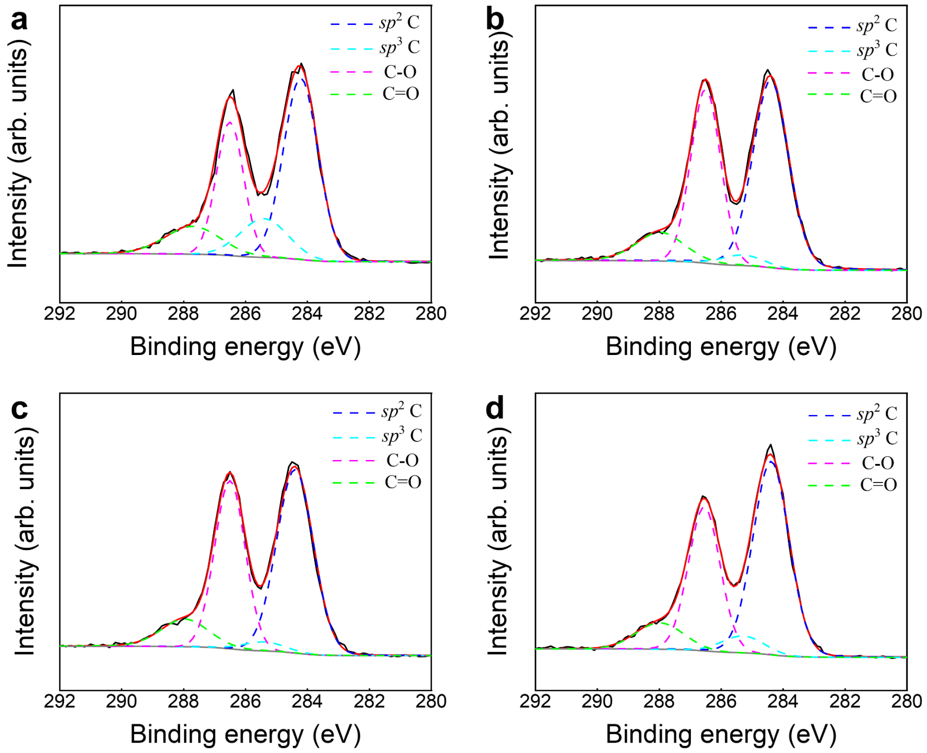

Supplementary Figure 33. XPS C1*s* fine spectra of products synthesized by industrial-scale LME at different running time. a, 2 days. **b,** 5 days. **c,** 30 days. **d,** 40 days.

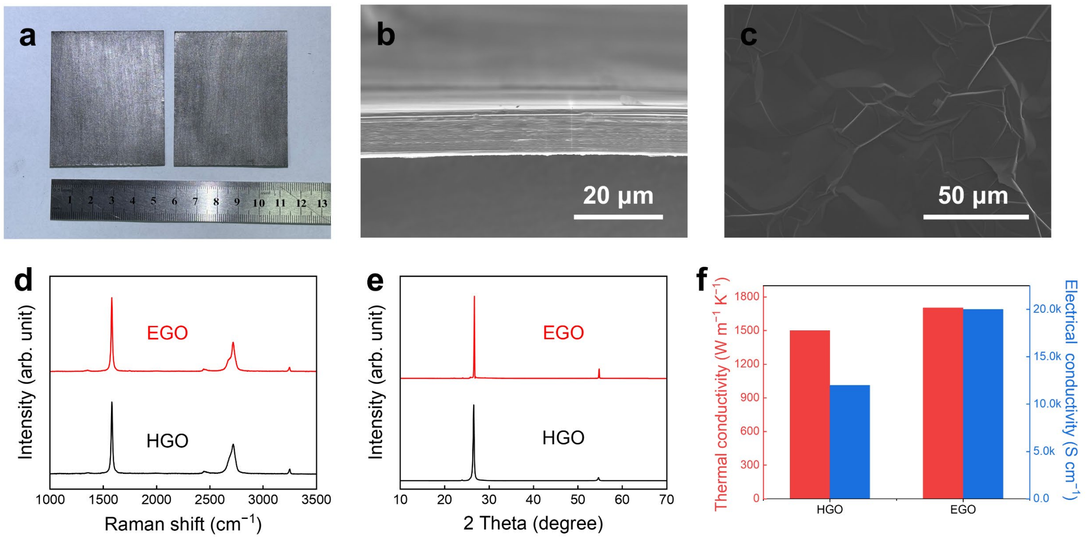

Fig. 34 The structure and properties of EGO-derived graphene films. a, Photograph of an EGO-derived graphene film. **b,** Cross-sectional scanning electron microscopy (SEM) image. **c,** SEM image of the surface. **d-f,** Comparisons of Raman spectra (**d**), X-ray diffraction patterns (**e**), and electrical and thermal conductivities (**f**) of EGO- and HGO-derived graphene films.

Supplementary Table 1 Reaction parameters of EC reactions with non-aqueous electrolytes and carbon content in the corresponding products.

NO.	Electrolytes	Reaction parameters	Carbon content
FGP-1	[BMIm][BF_4]	DC 20 V, 2 h	94.96%
FGP-2	$LiClO_4$(0.5 mol L^{-1}) + Polycarbonate	DC 20 V, 2 h	91.35%
FGP-3	Methanol + KPF_6 (KPF_6 saturated)	DC 60 V, 24 h	94.2%
FGP-4	Ethanol + KPF_6 (KPF_6 saturated)	DC 60 V, 24 h	86.57%
FGP-5	Methanol + $LiPF_6$ ($LiPF_6$ saturated)	DC 60 V, 24 h	96.10%
FGP-6	Ethanol + $LiPF_6$ ($LiPF_6$ saturated)	DC 60 V, 24 h	95.51%
GIC-1	[BMIm][BF_4]	DC 10 V, 2 h	93.5%
GIC-2	Ethanol + $NaPF_6$ ($NaPF_6$ saturated)	DC 60 V, 24 h	94.7%
GIC-3	Ethanol + KPF_6 (KPF_6 saturated)	DC 60 V, 24 h	95.6%

Supplementary Table 2 Properties of various anhydrous solvents and the corresponding T_f of SA-GIC-I in them.

Solvent	ε_r	Intersolubility	Reactivity	T_f
Water	80.1	Soluble	High	< 5s
Glycerin	46.53	Soluble	Low	490 s
Ethylene glycol	41.4	Soluble	Low	180s
Methanol	33	Soluble	High	100s
Ethanol	25.3	Soluble	High	150s
Propanol	20.28	Soluble	Low	320s
Isopropanol	20.18	Soluble	High	210s
Butanol	17.84	Soluble	Low	660s
Octanol	10.3	Slightly soluble	Low	1620s
Ethyl acetate	6.0814	Soluble	High	210s
Chloroform	4.8069	Insoluble	Inert	> 24 h
Carbon disulfide	2.6320	Insoluble	Inert	> 24 h
Carbon tetrachloride	2.24	Insoluble	Inert	> 24 h
Paraffin oil	2.05	Insoluble	Inert	> 24 h
Petroleum ether	1.84	Insoluble	Inert	> 24 h

Supplementary Table 3 Elemental composition (mass ratio) measured by EA for the GO synthesized by LME with different SA electrolytes.

SA concentration	C(wt.%)	N(wt.%)	H(wt.%)	S(wt.%)	O(wt.%)	C/O
0 wt.%	69.92	0.80	4.14	0.99	24.65	3.78
10 wt.%	62.54	0.81	3.78	0.93	31.94	2.61
20 wt.%	55.19	0.60	3.20	0.78	39.93	1.84
30 wt.%	47.16	0.60	2.98	0.93	47.14	1.34
40 wt.%	51.14	0.69	3.16	0.82	43.19	1.58
50 wt.%	58.27	0.78	3.60	1.00	36.47	2.13
60 wt.%	64.11	0.73	3.79	1.01	30.31	2.82

Supplementary Table 4 Review on the reaction parameters and properties of graphene/GO synthesized by the reported EC methods.

Raw materials	Electrolytes	Reaction conditions	Product properties	Ref.
Graphite flakes	1M H_2SO_4 in saturated $(NH_4)_2SO_4$ aqueous solution	constant current of 0.6 A for 24 h	I_D/I_G:NA<br>Thickness:66% monolayers<br>C/O:3.64<br>Lateral size: NA<br>Yield:37.6%	1
Graphite flakes	8 M $HClO_4$ aqueous solution	linear sweep voltammetry from ~0.2 to 1.4 V	I_D/I_G:0.75<br>Thickness: NA<br>C/O:9.81<br>Lateral size: NA<br>Yield: NA	2
Graphite flakes	11.6 M H_2SO_4 aqueous solution	constant current of 16 mA	I_D/I_G:1.18<br>Thickness:70% monolayers<br>C/O:4.07<br>Lateral size: 2.15 μm<br>Yield:155%	3
Pencil cores	1 M H_2SO_4 or H_3PO_4 aqueous solution	+1 V, 3–5 min; repeated alternation between ±7V	I_D/I_G:0.71<br>Thickness: 3–12 nm<br>C/O:NA<br>Lateral size: 1 to several μm<br>Yield: NA	4
Flexible graphite paper	98 and 50 wt. % H_2SO_4	step 1: +1.6 V for 20 min in 98%	I_D/I_G:NA<br>Thickness:95% monolayers<br>C/O:1.5–1.8	5

	aqueous solution	H_2SO_4; step 2: +5 V for 1 min in 50% H_2SO_4	Lateral size:1–5 µm<br>Yield:96%	
Graphite rods	1 M $(NH_4)_2SO_4$ (others: Na_2SO_4, $MgSO_4$, and H_2SO_4) aqueous solution	constant current of 1.0 A for 2 h	I_D/I_G:0.85<br>Thickness:1–3 layers 80%<br>C/O: NA<br>Lateral size: 0.5–2.5 µm 80%<br>Yield:>80%	6
Glassy carbon	50 wt.% H_2SO_4 aqueous solution	constant voltage of 5 V for 8 h	I_D/I_G:NA<br>Thickness: 1–5 nm, 90% 1–2 nm<br>C/O:1.4<br>Lateral size:<20 nm<br>Yield: NA	7
Graphite pellet	11.6 M $HClO_4$ aqueous solution	constant current density of 50 µA mg^{-1} for 17 h	I_D/I_G:NA<br>Thickness:1–2 nm 36%<br>C/O:3<br>Lateral size:10 µm<br>Yield: NA	8
Graphite foil	HBF_4 aqueous solution	constant current density (0.18A cm^{-2}) for 6 min with a cut-off	I_D/I_G:1.1<br>Thickness:62% monolayers<br>C/O:1.4<br>Lateral size: 0.04–1.78 mm<br>Yield: NA	9

		voltage of 14 V		
pyrolytic graphite sheets	2.0 M $HClO_4$ aqueous solution	a gradual increase from 0 to 8 V and then keeping at 8 V	I_D/I_G:1.04 Thickness: NA C/O:1.59 Lateral size: NA Yield:40%	10
Graphite paper	H_2SO_4 (>95%) and 0.1 M $(NH_4)_2SO_4$ aqueous solution	step 1: +2.2 V for 20 min in 95% H_2SO_4; step 2: +10 V for 5–10 min in 0.1 M $(NH_4)_2SO_4$	I_D/I_G:1.48 Thickness: monolayer, > 90% C/O:4.6 Lateral size:2–3 μm Yield:71%	11

Supplementary Table 5 Review on the reaction parameters and properties of GO synthesized by the reported chemical methods.

Ref.	Oxidation parameters	Oxidation degree	Yield
Our work-lab-scale	Step 1: 1.8 V; 98 wt.% H_2SO_4; 15 min<br>Step 2: 2.8 V; 30 wt.% H_2SO_4; ~1 cm liquid membrane; 1.9 mm min^{-1}	C/O：1.33 (EA)<br>2.0 (XPS)	181.2%
Our work-industrial-scale	Step 1: 5.0 V; 3.5 A; 98 wt.% H_2SO_4<br>Step 2: 5.5 V; 20 A; 50 wt.% H_2SO_4; ~1.5 cm liquid membrane; 1.5 mm h^{-1}	C/O：1.40 (EA)<br>2.08 (XPS)	156.9%
[12]	1 g Graphite; 30 ml H_2SO_4; 3g $KMnO_4$;<br>1h−35 °C; 30 mL H_2O 15 min−35 °C.	C/O: 2.0(XPS)	120%
[13]	Chemically expanded graphite:1g; H_2SO_4 30 mL; $KMnO_4$ 3g; 4h−35 °C.	Oxygen content: 27.5 wt.% (XPS)	100%
[14]	3 g CEG; 7.5~9 g $KMnO_4$:75 mL H_2SO_4 3h–35°C	C/O: 2.21 (XPS)	~100%
[15]	3 g Graphite; 9 g $KMnO_4$; 69 mL H_2SO_4; 1.5g $NaNO_3$	63 % oxidized carbon and 37% graphitic carbon	40%
[15]	3g Graphite; 360mL H_2SO_4; 40mL H_3PO_4 (9:1); 18.0 g $KMnO_4$; 50°C–12 h	69 % oxidized carbon and 31% graphitic carbon	193.3%
[16]	100 g Graphite; 50 $NaNO_3$; 300 g $KMnO_4$;<br>35 °C–30 min;4.6 liters water; 98 °C–15 min	C/O: 2.25 (EA)	188%
[17]	1 g Graphite (12000 mesh); 30 ml H_2SO_4; 3 g $KMnO_4$;	Oxygenated carbon atoms: 52.0%	152 ± 3%;

	40 °C–1 h; 50 ml water; 95 °C–15 min	(XPS)	
17	1 g Graphite (300−500 mesh); 30 ml H_2SO_4; 3 g $KMnO_4$; 40 °C–3 h; 90 ml water; 40 °C–1 h	Oxygenated carbon atoms: 55.0% (XPS)	113 ± 2%;
17	0.5 g Graphite (80 mesh); 30 ml H_2SO_4; 1.5 g $KMnO_4$; 40 °C–5 h; 90 ml water; 40 °C–1 h	Oxygenated carbon atoms: 57.6% (XPS)	96 ± 2%;
18	1 g Graphite; 23 ml H_2SO_4; 3g $KMnO_4$; 40 °C−0.5 h; 50 mL H_2O 95 °C−15 min.	Oxygenated carbon atoms: 50.4% (XPS)	171 ± 4%
19	3 g Graphite (325 mesh); 70 ml H_2SO_4; 9 g $KMnO_4$; 40 °C–0.5 h; 150 ml water; 95 °C–15 min	C/O: 2.36(XPS)	92 ± 3%

Supplementary note 1. EC oxidation of graphite and GIC in non-aqueous electrolytes

To confirm the indispensability of water for GO synthesis, we studied the EC reactions of graphite (here, flexible graphite paper, FGP) and GIC in different kinds of non-aqueous electrolytes. The reaction parameters and oxidation degree of the corresponding products are shown in **Supplementary Table 1**. Considering the inevitable absorption of oxygen, here we used carbon content measured by XPS to show the oxidation degree of the products.

As shown in **Supplementary Figure 1,** the FGP anodes were partly exfoliated after EC reactions using [BMIm][BF_4] and $LiClO_4$/Polycarbonate as electrolytes (experiments No. 1 and 2). For the other four kinds of electrolytes, only slight swelling of the anode was observed after 24 h EC reactions with voltage as high as 60 V. As shown in **Supplementary Figure 2,** the survey XPS spectra and high-resolution C 1*s* XPS spectra of the six products show very weak oxygen related peaks (O 1*s* peak @ ~540 eV, C=C peak @ ~284.6 eV, C-C peak @ ~285.4 eV, C–O peak @ ~287.0 eV, and carboxylate carbon @ ~289.0 eV). The obvious π-π* peaks (~291.5 eV) prove that the conjugated structure of the carbon lattices in these products is nearly intact during EC reaction.

In addition to the findings from the FGP, the properties of reaction products from SA-GIC-I shows nearly the same characters. Though some reactions in non-aqueous electrolytes (experiments No. 7) result in exfoliation of anodes, their products show no obvious character of oxidation. These results suggest that it is difficult to generate covalent functional groups on carbon lattices neither in graphite nor in GIC by EC reaction in non-aqueous electrolytes.

Supplementary note 2. In-situ Raman investigation on DIWA of SA-GIC-I in air with different humidity

As shown in **Supplementary Figure 6 and Supplementary Table 6**, air environments with set RH were created in a sealed PTFE reactor containing P_2O_5 dry powder or different saturated salt solutions at 24 ± 0.5 °C. [20] The reactor was sealed by a thin PE membrane, which prevented the matter exchanging between the reactor and the outer atmosphere without affecting Raman spectroscopy testing.

Supplementary Table 6 Modulating relative humidity (RH) of air by using different chemical reagents in reactor

RH of air	Chemical reagent in reactor
0%	P_2O_5 (dry powder)
11%	LiCl (supersaturated aqueous solution)
33%	$MgCl_2$ (supersaturated aqueous solution)
53%	$Mg(NO_3)_2$ (supersaturated aqueous solution)
70%	$SrCl_2$ (supersaturated aqueous solution)
100%	Pure water

SA-GIC-I samples were synthesized by EC intercalation of FGP anodes (slice dimensions: 30 mm length × 10 mm width × 0.2 mm thickness) in 98 wt.% SA for 15 min under voltage of 1.8 V. Before testing, the sealed reactor was kept at a constant temperature (24 ± 0.5 °C) for more than 24 hours to obtain a stable humidity environment. Then freshly synthesized SA-GIC-I sample (20 mm × 10 mm) was placed on the dais area in the reactor. After that, the reactor was sealed again and placed on the sample holder of the Raman spectrometer as quickly as possible.

The Raman spectra were measured using 532 nm laser with an integration time of 5 s and different time intervals in air with different humidity (**Supplementary Figure 7**). A typical characteristic of DIWA is the red shift of Raman G peak from 1637 cm^{-1} (stage-I) to 1617 cm^{-1} (stage-II) [11,21,22]. In fully dried air atmosphere, the intercalation state of SA-GIC-I sample kept stable for more than 5 days. However, when the RH of air increased to 11%, deintercalation occurred obviously. The higher RH, the

quicker DIWA rate.

Supplementary note 3. Stability of SA-GIC-I in anhydrous solvents

SA-GIC-I samples were synthesized by EC intercalation of FGP anodes (slice dimensions: 25 mm length × 10 mm width × 0.2 mm thickness) in 98 wt.% SA for 15 min under voltage of 1.8 V. Freshly synthesized SA-GIC-I slices were immersed into different kinds of anhydrous solvents. Since the color change from blue to gray is the typical characteristic of deintercalation of SA-GIC-I, the time needed for the fully fading of blue color of the GIC surface (T_f) was used to evaluate the deintercalation rate (DR). The smaller T_f, the higher DR. The properties of the various anhydrous solvents, including relative dielectric constant (ε_r), and intersolubility and reactivity with SA, and the corresponding T_f of SA-GIC-I are listed in **Supplementary Table 2**.

Generally, due to the positive correlation between ε_r and polarity of the solvent, SA-GIC-I is stable in nonpolar solvents ($\varepsilon_r < 5$) but unstable in polar solvents ($\varepsilon_r \geq 5$). Five kinds of nonpolar solvents [chloroform ($CHCl_3$), carbon disulfide (CS_2), carbon tetrachloride (CCl_4), petroleum ether (PE), paraffin oil (PA)] were found to be insoluble and chemically inert in SA (both with and without water). When immersing in these solvents, SA-GIC-I slice can keep stable for more than 24 h, which allows the use of these solvents for "oil sealing".

Polar solvents used in this study are all common solvents. Most of them are miscible with SA and can react with SA to different extent. As shown in **Supplementary Figure 8**, the DR of SA-GIC-I increases sharply with the increase in polarity for most solvents. However, several solvents do not follow this trend such as ethyl acetate and glycerin. The former has low ε_r but high DR, while the latter has high ε_r but relatively low DR. This might be due to their different chemical reactivity with SA. Higher reactivity may facilitate deintercalation. For instance, propanol and isopropanol have nearly the same ε_r, but isopropanol is more reactive with SA. The DR of SA-GIC-I in isopropanol is 1.5 times higher than that in propanol.

Supplementary note 4. Controlled synthesis of SA-GIC samples with different intercalation stages

EC synthesis of SA-GIC have been studied for long time[23-26]. Generally, SA-GIC anodes with different stages have different electrode potentials. Thus, SA-GIC samples with different intercalation stages can be synthesized by controlling the applied voltage.

In this study, by serial experiments, the Voltages to synthesize stage-I, stage-II, and stage-III SA-GIC using FGP as anode were found to be 1.8 V, 1.4 V and 1.1 V, respectively. The setup for EC intercalation is shown as follows.

Anode: FGP slice with dimension of 15 cm (length) × 0.8 cm (width) × 0.2 mm (thickness)

Cathode: Pt wire with dimension of 15 cm (length) ×0.5 mm (diameter)

Electrolyte: 98 wt.% SA

Reaction time: 15 min

As shown in **Supplementary Figure 9a**, there are obvious differences in the color of SA-GIC samples synthesized under different Voltages. The color of the sample obtained at 1.8 V is blue, which is the typical feature of SA-GIC-I. However, the color of the samples obtained at 1.4 V and 1.1 V is gray and blackish, respectively. The XRD patterns of the three samples are shown in **Supplementary Figure 9b**, which are consistent with the standard spectra of GIC with corresponding stages[27,28]. This is the direct evidence of controlled synthesis of SA-GIC with different stages. The Raman spectra of these three samples are shown in **Supplementary Figure 9c**. As has discussed in **supplementary note 3**, the G peak of SA-GIC-I locates at 1637 cm^{-1}. The obvious blue shift compared with that of graphite (1580 cm^{-1}) is caused by the strong doping effect of intercalated SA[29-32]. Moreover, the doping degree decreases when increasing the intercalation stage to II and III. Thus, the G peak of SA-GIC-II is red shifted to 1617 cm^{-1}. Besides further red shift, the G peak of SA-GIC-III splits into two peaks at 1587 cm^{-1} and 1609 cm^{-1}, which correspond to the vibration modes of the pristine graphite layer and the graphite layer surrounding with SA on one side,

respectively[23].

Supplementary note 5. Emulsion electrolysis

To synthesize water-in-oil colloidal emulsion, 10 g water and 100 g PA was first mixed using Span 85 as surfactant (5 g, pre-dissolved in PA) [33,34], as shown in **Supplementary Figure 11a**. After 10 minutes of high shear dispersion (FLUKO FM30D), a semitransparent uniform dispersion was obtained (**Supplementary Figure 11b**), which shows obvious Tyndall effect. The average particle size of the colloidal particles in this emulsion was determined to be about 247.4 nm by a laser particle size analyzer (**Supplementary Figure 11c**). As a result, the mean water amount in one colloid particle was no more than 6.34×10^{-6} ng.

The emulsion electrolysis device is shown in **Supplementary Figure 10a**. Before emulsion electrolysis, SA-GIC samples with different stages were first synthesized as shown in **Supplementary note 4**. Notably, during EC intercalation, the surface of 98% SA was covered by a PA layer (thickness ~10 mm), which prevented the water absorption of SA from air. After EC intercalation, SA was pumped out from the bottom and extra PA was added into the reactor from the upper inlet simultaneously at the same rate. Thus, the upper surface level of PA kept constant and the SA-GIC samples were fully protected from contacting with air during the whole process.

As shown in **Supplementary Figure 10b**, for emulsion electrolysis, the distance between Pt cathode and GIC anode was decrease to 5 mm and the output voltage was set as 10 V. Then, 200 μL of water containing emulsion was added drop-wisely into the reactor with a pipette gun. By such treatment, trace amount of water was introduced into the EC reaction. The EC reaction of SA-GIC anode can only occur when ultra-small water droplets, which were encapsulated by surfactant layers in water-in-oil colloid particles, spread to the surface of the SA-GIC anode. After reaction for 6 h, the products achieved were lifted out from the reactor and sonicated in water for 20 min. The obtained dispersions were centrifuged at 450 g for 3 times (15 min for each centrifugation), then the supernatants were collected for subsequent characterization.

As shown in **Supplementary Figure 12a**, the dispersions of emulsion electrolysis products synthesized from SA-GIC with different intercalation stages have obvious

difference in color. They are brown, yellow, and gray for SA-GIC-I, SA-GIC-II, and SA-GIC-III as raw material, respectively, suggesting the different oxidation degree of the products[35,36]. The XPS and FTIR spectra indicate that these products have similar types of oxygen-containing groups, but the oxidation degrees are different. The C/O ratios of products measured by XPS are 1.8, 3.6, and 8.8 for SA-GIC-I, SA-GIC-II, and SA-GIC-III as raw materials, respectively (**Supplementary Figure 12b–c**). TG characterization further confirm the differences in their degree of oxidation degree (**Supplementary Figure 12d-e**).

Supplementary note 6. Device for in-situ microscopic observation and Raman spectroscopic investigation

Supplementary Figure 13 shows the device for in-situ microscopic observation and Raman spectroscopic investigation. It involves a Raman spectrometer (532 nm laser, 50 × objective lens), electrochemical workstation and a homemade in-situ EC reactor. The electrolytic tank is the same as that used for in-situ Raman investigation on DIWA of SA-GIC-I in air with different humidity. The cathode and anode holders are both made of thin Pt wires, which were set on the dais part of the tank. The anode holder was used for fixing the SA-GIC-I slice and making it connecting to the electrochemical workstation. Both anode and cathode were immersed in PA to prevent them from contacting with air. For EC reaction, a small amount of electrolyte (30% SA aqueous solution) was added into the space between cathode and anode to connect them, building a complete circuit.

Supplementary note 7. In-situ experimental investigation of DIWA and OWE of SA-GIC-I

We have found that water is the most critical factor in controlling DIWA and OWE during the electrochemical oxidation process of SA-GIC. Consequently, we investigated how the behaviors of these two processes changed by altering water-related control parameters. This investigation will help us identify the key conditions to maintain the equilibrium between these two processes.

Before examining the influence of concentration and volume of the electrolyte – two parameters that are directly related to water, we first investigated the impact of applied voltage on DIWA and OWE. This is primarily due to the fact that the charged intercalant is significantly influenced by the applied potential, which may result in the change of varying trends caused by water. In-situ investigations of the effects of voltage were conducted using the following parameters:

1. Experiment parameters:

(1) Device: In-situ EC reactor

(2) Dimension of anode: 1 cm (length) × 1 cm (width) × 0.1 mm (thickness)

(3) Parameters for EC oxidation: Electrolyte: 30 wt.% SA; Electrolyte amount added:100 μL; **Voltages (controlling factor): 1.0 V, 1.3 V, 1.6 V and 1.9 V**.

2. Experiment parameters:

(1) Device: In-situ EC reactor

(2) Dimension of anode: 1 cm (length) × 1 cm (width) × 0.1 mm (thickness)

(3) Parameters for EC oxidation: Electrolyte: 30 wt.% SA; Electrolyte amount added:100 μL; **Voltages (controlling factor): 2.2 V, 2.4 V, 2.6 V, 2.8 V, 3.0 V and 3.2 V**.

The intercalated species in SA-GIC are primarily negatively charged $(H_2SO_4)_x(HSO_4^-)_y$. When SA-GIC anode was charged, the attraction force between the intercalated species and the positively charged graphene lattices increased with the increase of applied voltage, which resulted in the decrease of deintercalation rate (**Supplementary Figure 14**). When the voltage exceeded the threshold, the

deintercalation was suppressed and the oxidation of graphite occurred. However, once graphite was oxidized, it became insulating and hydrophilic with an increase in interlayer distance, which reduced the attraction towards ions and facilitates water diffusion. In most instances, DIWA demonstrated a faster rate than OWE, suggesting that deintercalation within the reaction zone occurred before oxidation. Increasing the oxidation voltage improved the balance between DIWA and OWE. When the applied voltage increased to 2.2-3.2 V, we achieved a uniformly oxidized graphite **Supplementary Figure 15**.

We then used the following parameters for in-situ investigations on the effect of electrolyte concentration and volume on DIWA and OWE. Experimental results show that an increase in water content or volume significantly accelerated DIWA rate. When the rate of DIWA was faster than the rate of OWE, non-uniform oxidation occurred (**Fig. 2e**).

3. Experiment parameters:

(1) Device: In-situ EC reactor

(2) Dimension of anode: 1 cm (length) × 1 cm (width) × 0.1 mm (thickness)

(3) Parameters for EC oxidation: Voltages: 2.8 V; Electrolyte amount added:100 μL; **Electrolyte (controlling factor): 0 wt.%, 10 wt.%, 20 wt.%, 30 wt.%, 40 wt.%, 50 wt.%, 60 wt.% SA aqueous solutions**.

4. Experiment parameters:

(1) Device: In-situ EC reactor

(2) Dimension of anode: 1 cm (length) × 1 cm (width) × 0.1 mm (thickness)

(3) Parameters for EC oxidation: Electrolyte: 30 wt.% SA; **Electrolyte amount added (controlling factor): 100 μL, 200 μL, 300 μL, 400 μL, 500 μL, 600 μL**; Voltages: 2.5 V

Supplementary note 8. Detailed process of LME method

The LME device mainly consists of two key subsystems: (1) reactor and fluid transport subsystem, and (2) electrical and automatic control subsystem. The latter was used to control the collaborative work of various fluid transport components in the reactor according to the set program.

As mentioned in the main text, beside proper voltage applied between anode and cathode and proper concentration of SA electrolyte, the following two factors are essential for sufficient and efficient GO synthesis: (1) preventing SA-GIC-I anode from contacting with humid air by oil sealing, and (2) decreasing the contact area between SA-GIC-I anode and aqueous electrolyte by forming liquid membrane (LM). The basic process to realize such control is schematically shown in **Supplementary Figure 18**, which involves EC intercalation of FGP to form SA-GIC-I (**step I**) and EC oxidation of SA-GIC-I to from graphite oxide by LME (**step II**). Both reactions were carried out in one reactor but with different working liquids, as shown in **Supplementary Table 7**.

Supplementary Table 7. Classification and properties of working liquids for LME.

No.	Name	Function	Typical density	Reagent
1	Light oil	Oil sealing above electrolyte layer	≤ 0.86 g mL^{-1}	PA
2	Concentrated SA	Reagent for EC intercalation	~1.84 g mL^{-1}	98 wt.% SA
3	Dilute SA	Reagent for EC oxidation	1.0~1.2 g mL^{-1}	30 wt.% SA
4	Heavy oil	Oil sealing beneath electrolyte layer	1.2~1.6 g mL^{-1}	CCl_4

In step I, working liquids 1 and 2 were used as shown in **Supplementary Figure 18a-c**. The 98 wt.% SA was the reagent for EC intercalation, and PA was used to protect the SA and SA-GIC-I from contacting with humid air. In step II, working liquids 3 and 4 were added from the bottom of the reactor as shown in **Supplementary Figure 18d-f**, generating a thin layer of 30 wt.% SA (i.e. LM) sandwiched between the PA layer above and the CCl_4 layer below. Thus, only the contacted small part of anode was transformed into graphite oxide, while the other part of anode that was sealed by PA

remained was not changed. The whole anode was gradually oxidized with the rising of LM, and the rising rate was controlled by the adding speed of CCl_4. The LME stopped when the PA was fully flowed out, and in this case, the whole SA-GIC-I anode was fully transformed into graphite oxide (**Supplementary Figure 18f**). **Supplementary Figure 19** gives the basic components of a homemade LME device and **Supplementary video S5** clearly shows the synthesis process of graphite oxide by the LME device.

In the above process, PA (working liquid 1) and CCl_4 (working liquid 4) were recycled. There are a small amount of consumptions of concentrated SA (working liquid 2) and diluted SA (working liquid 3) because of their participation in EC intercalation and oxidation, respectively. However, compared to the traditional chemical oxidation technology, the SA consumption in LME technology is negligible. For instance, only 1.1 ml SA was used for the production of 1.0 g pure GO, which is almost 20 times less than the Hummers' method.

Supplementary note 9. GO synthesis by LME with different LM thicknesses and rising speeds

Parameters used for GO synthesis by LME:

(1) Device: LME reactor

(2) Dimension of FGP anode: 15 cm (length) × 0.8 cm (width) × 0.2 mm (thickness)

(3) Parameters for EC intercalation: Electrolyte: 98 wt.% SA; Voltage: 1.8 V; reaction time: 15 min.

(4) Parameters for EC oxidation: Electrolyte: 30 wt.% SA; Voltages: 2.8 V； **LM thickness (controlling factor): 3 mm – 18 mm (1 mm intervals), ∞ (No heavy oil); LM rising speed (controlling factor): 1 mm min^{-1}, 1.9 mm min^{-1}, 2.7 mm min^{-1}, 3.5 mm min^{-1}**.

Similar to the results of in-situ experiments, efficient oxidation in the LME reactor requires maintaining the dynamic equilibrium between DIWA and OWE. Therefore, when LME was used to synthesize GO, not only the thickness but also the rising speed of LM should be considered. We studied the influence of these two parameters on the synthesis of GO by LME. It was found that thinner LM was beneficial for improving the uniformity of oxidation and the synthesis of monolayer GO (**Supplementary Figure 20 and 21**), but the overall oxidation speed of the electrode was reduced. In addition, the faster the LM rising speed, the shorter the EC oxidation time, leading to insufficient oxidation (**Supplementary Figure 22**). In particular, when using a thin LM and a high rising speed simultaneously, severely insufficient oxidation occurred. In this case, only the exterior of the anode was oxidized while the interior was not oxidized. Therefore, a larger amount of graphene and graphite flakes were found in the GO dispersion after exfoliation.

Based on the above investigations, optimized parameters for GO synthesis by LME were determined as: Electrolyte: 30 wt.% SA; LM thickness: 8 mm; LM rising speed: 1.9 mm min^{-1}; Voltage: 2.8 V.

Supplementary note 10. Controlling the lateral size distribution of GO by LME

Experiment parameters:

(1) Device: LME reactor

(2) Dimension of FGP anode: 15 cm (length) × 0.8 cm (width) × 0.2 mm (thickness)

(3) Parameters for EC intercalation. Electrolyte: 98 wt.% SA; Voltage: 1.8 V; reaction time: 15 min.

(4) Parameters for EC oxidation: Electrolyte: 30 wt.% SA; LM thickness: 8 mm; LM rising speed: 1.9 mm min^{-1}; **Voltages (controlling factor): 2.5 V, 2.8 V, 3.5 V and 4 V**.

SEM characterizations clearly show that the sample's average lateral size can be finely tuned by the voltage (**Supplementary Figure 24**). It is worth noting that large GO (~50 μm) can be synthesized at a relatively low voltage (2.5 V), which have never been achieved by other EC methods[5,7,11,37-45] (**Supplementary Figure 25**). Actually, large GO is also difficult to synthesis even by the Hummers' method[14,46,47]. Moreover, these large GO synthesized by LME are still monolayer dominated, with a monolayer ratio over 96% (**Supplementary Figure 26**). These results confirm that the lateral size of GO can modulated by simply adjusting the oxidation voltage of LME, while other properties changed slightly.

Supplementary note 11. Controlling the oxidation degree of GO by LME

Experiment parameters:

(1) Device: LME reactor

(2) Dimension of FGP anode: 15 cm (length) × 0.8 cm (width) × 0.2 mm (thickness)

(3) Parameters for EC intercalation. Electrolyte: 98 wt.% SA; Voltage: 1.8 V; reaction time: 15 min.

(4) Parameters for EC oxidation: Voltages: 2.8 V; LM thickness: 8 mm; LM rising speed: 1.9 mm min^{-1}; **Electrolytes (controlling factor): 0 wt.%, 10 wt.%, 20 wt.%, 30 wt.%, 40 wt.%, 50 wt.%, and 60 wt.% SA**.

Supplementary Figure 27, **Supplementary Figure 28** and **Supplementary Table 3** indicate that the oxidation degree of GO products could be efficiently tuned by changing the concentration of SA electrolyte. In general, the oxidation degree of the samples increased first and then decreased with increasing electrolyte concentration, reaching a maximum value at 30 wt.%. The monolayer ratio of the samples followed the same trend as the oxidation degree, but the monolayer ratio was still higher than 80% even for the samples with the lowest oxidation degree (**Fig. 4c**). Importantly, only little change in lateral size was observed along with the change of oxidation degree, as shown in **Supplementary Figure 29**.

Supplementary note 12. Industrial-scale production of GO by LME

In comparison to lab-scale synthesis, the following three requirements are essential for industrial production of GO: (1) highly automated equipment, (2) long-term operational stability, and (3) unwavering product quality. Based on these requirements, we designed our industrial equipment. As illustrated in **Supplementary Figure 30**, the entire system is compartmentalized into five key sections: the automatic control system (labeled A), the power supply system (labeled B), the conveyor system (labeled C), the reaction system (labeled D), and the SA circulation system (labeled E). The entire equipment size is 160 cm (length) × 130 cm (width) × 190 cm (height).

Through the coordination of the aforementioned five systems, continuous oxidation of FGP with a width of 1 m has been realized. The following are the reaction parameters:

(1) Device: industrial-scale LME reactor

(2) Dimension of FGP anode: 85 m (length) × 100 cm (width) × 0.2 mm (thickness).

(3) Density of FGP anode:1.54 g cm^{-3}.

(3) Parameters for EC intercalation: Electrolyte: 98 wt.% SA; Voltage: 5.0 V; Current: 3.5 A.

(4) Parameters for EC oxidation: Electrolyte: 50 wt.% SA; LM thickness: 15 mm; LM rising speed: 9 cm h^{-1}; Voltages: 5.5 V; Current: 20 A.

(5) Supplement of various solutions: H_2O: 2 kg day^{-1}; SA: 2 kg day^{-1}; Heavy perfluoropolyether and light perfluoropolyether: < 20 g day^{-1}.

After the oxidation was completed, the graphite oxide obtained was cleaned four times by using an industrial-grade filter press. Then, it was subjected to ultrasonication for exfoliation to get GO dispersion. Extensive SEM measurements revealed that the product has an average lateral size of ~1 μm (**Supplementary Figure 31a–b**). The statistical analyses of 150 samples indicated that 93.75% flakes are monolayers, and over 99% are less than 3 layers (**Supplementary Figure 31c−d**).

The chemical composition of industrial-scale EGO was analyzed by FTIR

(**Supplementary Figure 32a**) and XPS (**Supplementary Figure 32b–c**), and the results are similar to those of typical HGO. Combustion elemental analysis results showed that the elemental composition (mass ratio) of EGO is: C (48.58%), O (46.38%), H (2.75%), N (0.08%), and S (2.22%). The calculated C/O atomic ratio is approximately 1.40, which is comparable to those of reported lab-scale HGO samples. Moreover, the industrial-scale EGO products showed similar Raman spectra with HGO (**Supplementary Figure 32d**). Further investigations using UV-Vis and TGA confirmed their high oxidation degree (**Supplementary Figure 32e–f**).

Overall, the number of layers, lateral size and chemical composition of industrial-scale EGO are on par with those synthesized by the Hummers' method at lab-scale.

Finally, we assessed the yield of the industrial-scale LME by running the equipment continuously for 24 hours to collect the product. The total mass of the product obtained was 2087 g. Therefore, the yield of industrial-scale EGO can be calculated as follows:

$$
\begin{aligned}
Yield\ (\%) &= \frac{Product\ mass}{Raw\ materials\ mass} \times 100\% \\
&= \frac{2087g}{Width \times thickness \times length \times density \times 2} \times 100\% \\
&= \frac{2087g \times 100\%}{100\ cm \times 0.02\ cm \times 9\ cm\ h^{-1} \times\ 24\ h \times 1.54\ g\ cm^{-3} \times 2} \times 100\% \\
&= 156.85\%
\end{aligned}
$$

Note that this high yield can also rival that of the Hummers' method at lab-scale, demonstrating that our LME method is capable of achieving GO with a high oxidation degree at industrial-scale.

The long-term stability of the LME reaction was also evaluated. Given that a whole roll of graphite paper can be used for 40 days, we selected four specific time points (2, 5, 30, and 40 days) to conduct XPS tests on the product. Almost the same C1s fine spectra (**Supplementary Figure 33**) of all products reveal that they have the same fundamental bonding structure, suggesting the high stability of LME.

Supplementary note 13. A comparative analysis of LME method and the Hummers' method on industrial-scale

In the Hummers' method, the oxidation reaction of flake graphite (80 mesh) was conducted in a 1 m^3 enamel reaction kettle. For safety purpose, 2/3 of the total volume of the reactor was utilized. Initially, 150 kg of concentrated SA (98wt.%) and 5 kg of flake graphite were added to the reactor, followed by stirring for 2 hours to ensure thorough mixing. Subsequently, 15 kg of potassium permanganate was added to the reactor using an automatic feeder. This gradual feeding process, lasting nearly 10 hours, was necessary to prevent excessive accumulation of potassium permanganate on the surface, potentially causing an explosion. This extended duration was attributed to the lower density of potassium permanganate (2.7 g cm^{-3}) compared to concentrated SA and its limited solubility in SA. Throughout the whole oxidation process, cooling water was continuously fed to maintain a low temperature. After the addition of potassium permanganate, a sequential 12-hour agitation period was necessary to ensure the uniformity of the reaction. During this 12-hour homogeneous reaction, the flake graphite was only converted into pristine graphite oxide[27] (PGO). Therefore, approximately 500 kg of water was added and agitated to initiate water-enhanced oxidation. Upon cooling of the reaction system, the resulting graphite oxide was collected from the kettle for subsequent cleaning. The entire reaction typically lasted for two days, yielding 8 kg pure GO. Therefore, the industrial-scale yield of the Hummers' method is around 160%.

Since the equipment used in the subsequent cleaning process was the same, we compared the two methods based on the normalization of the aforementioned equipment input. Each set of enamel reaction kettle and the supporting steel frame costs around ¥ 100,000, while each set of industrial-scale LME equipment costs about ¥ 40,000. Therefore, given the same (¥ 100,000) asset input, the efficiencies of the two methods are 5217.5 g day^{-1} and 4000 g day^{-1}.

In addition to production efficiency, the production cost is another very important factor to evaluate an industrial-scale production process. The raw materials consumption of the two methods are shown as follows:

Supplementary Table 8 The cost of raw materials used in the two methods

Raw materials	Hummers' method	LME method	Price
Flake graphite	1 kg	Unused	¥ 6 kg^{-1}
FGP	Unused	1 kg	¥ 36 kg^{-1}
H_2SO_4	30 kg	1 kg	¥ 1 kg^{-1}
$KMnO_4$	3 kg	Unused	¥ 18 kg^{-1}
Water	100 kg	1 kg	¥ 0.004 kg^{-1}
H_2O_2	2 kg	Unused	¥ 0.95 kg^{-1}
Perfluoropolyether	Unused	0.010 kg	¥ 500 kg^{-1}

Based on above data, the cost of raw materials required to produce 1 kg of GO can be calculated. In the Hummers' method, the total cost of the raw materials is ¥ 57.69, whereas for the LME method, the cost is reduced to ¥ 26.77.

Supplementary Table 9 Energy consumption of the two methods (two days as a cycle)

Energy-consuming component	Hummers' method	LME method	Time
EC reaction power supply	Unused	0.1275 kW	48 h
Control and transmission systems	Unused	0.8 kW	48 h
Stirring motor	8 kW	Unused	48 h
Cooling-water machine	3 kW	Unused	12 h
Automatic feeder	0.5 kW	Unused	10 h

Another significant component of production cost is energy consumption. For the LME method, the precise control of the water diffusion process eliminated the need for excessive applied voltage. According to manufacturing parameters, we calculated the energy consumption per kilogram of GO. For the Hummers' method, the energy consumption (E_H) is:

$$E_H = \frac{8kW * 48h + 3kW * 12h + 0.5kW * 10h}{8kg} = 53.125\ kWh\ kg^{-1}$$

In contrast, the energy consumption (E_L) in LME is:

$$E_L = \frac{0.1275kW * 48h + 0.8kW * 48h}{2.087kg} = 21.332\ kWh\ kg^{-1}$$

Considering the unit electricity price in Shenzhen (¥ 0.9298 kW^{-1} h^{-1}), the energy cost for producing one kilogram of GO via Hummers' method was calculated to be ¥49.39, whereas the energy cost for LME is ¥19.83.

Treatment cost of wastewater is the third significant component of production cost of GO. In the Hummers' method, the water consumption for cleaning 1 kg of GO ranges from 400-1000 kg at the lab-scale, but it can be reduced to 200 kg in industrial production through the use of press filtration. In contrast, in the LME process, cleaning 1 kg of EGO product only requires a maximum of 50 kg of water as SA is minimally consumed. In terms of wastewater treatment, the wastewater from the LME process mainly consists of approximately 10 wt.% SA solution, with a treatment cost of ¥ 2,000 per ton. However, the wastewater in the Hummers' method is a SA solution containing Mn^{2+}, and the treatment cost is generally more than ¥ 5,000 per ton. As the cost of wastewater treatment reflects the level of pollution, it is considered as a quantitative measure of eco-friendliness. Calculations show that the cost of wastewater treatment in the LME process is approximately 1/10 of that in the Hummers' method, suggesting the much better eco-friendliness of LME method.

Considering the above three main contributors into the production cost, raw materials, energy consumption and treatment of wastewater, the cost ratio associated with the production processes of the two methods is:

$$\begin{aligned} Cost\ ratio &= \frac{LME\ method\ cost}{Hummers'\ method\ cost} \\ &= \frac{26.77 + 19.83 + 100}{57.69 + 49.39 + 1000} \\ &= 0.1324 \end{aligned}$$

And, the cost of GO is 0.146 ¥ g^{-1} and 1.11 ¥ g^{-1} for LME method and the Hummers' method respectively.

Since the worker's responsibilities extend beyond producing GO, it is challenging to allocate the employee's cost to a single unit of GO. Consequently, this portion of the

cost, which is difficult to quantify, was omitted in our calculations. However, the automation inherent in the LME process is likely to greatly reduce labor costs, thereby further enhancing its cost advantages.

The LME method is also significantly safer than the Hummers' method. We conducted an analysis of various risks in the production process according to the requirements of the Safety Checklist Analysis (SCL) [48]. Each risk was scored, with high risk assigned a value of 0, low risk a value of 10, and no risk a value of 20. The risks and their corresponding levels in the production process are listed in **Supplementary Table 10**.

Supplementary Table 10 Safety risks in the production process of the two methods

Safety risks	Hummers' method	LME method
Oxidizing agent storage risk	High risk	No risk
H_2SO_4 leakage risk	Low risk	Low risk
Explosion risk during reaction	High risk	No risk
Thermal runaway risk	High risk	No risk
Mechanical injury risk	High risk	Low risk

Based on the aforementioned calculation rules, the LME achieves a score of 80, whereas the Hummers' method only achieves a score of 10. Consequently, we can determine the safety ratio between these two methods. Considering that the calculated results may not fully capture the realities of actual production, we have conducted long-term observations of the production outcomes for both reactions. Each graphite roll is approximately 85 m in length, allowing the LME to run continuously for up to 40 days. Throughout this 40-days period, only one dilute SA circulating pump was obstructed in the two sets of equipment. The safety risks associated with this obstruction can be easily mitigated through manual dredging, which costs less than ¥ 100. In contrast, two explosions arising from uneven mixing of potassium permanganate occurred within one month during the Hummers' production process, leading to raw material losses and cleanup expenses exceeding ¥ 2500. Thus, the LME offers a much safer alternative to the Hummers' method for the industrial-scale production of GO, free from safety hazards.

Finally, compared to the Hummers' method, LME offers the advantage of high controllability. The Hummers' method often requires adjustments of the reaction temperature or the amount of potassium permanganate in order to regulate the oxidation degree[12]. Controlling the size of the GO flakes often requires the change of the graphite raw material and oxidation parameters. In contrast, for the LME method, we can easily control the basic properties of GO by fine-tuning the voltage or electrolyte parameters without additional steps.

Supplementary note 14. Fabrication of thermally conductive graphene films

To demonstrate the potential applications and advantages of our EGO, as an example, we fabricated thermally conductive graphene films as reported previously[49]. For comparison, we also fabricated thermally conductive graphene films under the same conditions by using HGO as raw materials. As shown in Fig. S34a-c. the EGO-derived graphene films show a smooth surface and highly compact layered structure. The more asymmetrical Raman 2D peak and narrower XRD (002) peak of EGO-derived graphene film suggest much better crystallinity and ordering than those of HGO-derived graphene film (Fig. S34d, e). As a result, EGO-derived graphene film exhibits much higher electrical and thermal conductivities (~20000 S cm^{-1} and ~1700 W $m^{-1}K^{-1}$) than those of HGO-derived graphene film (Fig. S34f).

Supplementary References

1 Yu, P. *et al.* Mechanically-Assisted Electrochemical Production of Graphene Oxide. *Chem. Mater.* **28**, 8429-8438, (2016).

2 Gurzęda, B. *et al.* Synthesis of graphite oxide by electrochemical oxidation in aqueous perchloric acid. *Carbon* **100**, 540-545, (2016).

3 Lowe, S. E. *et al.* Scalable Production of Graphene Oxide Using a 3D-Printed Packed-Bed Electrochemical Reactor with a Boron-Doped Diamond Electrode. *ACS Appl. Nano Mater.* **2**, 867-878, (2019).

4 Liu, J. *et al.* A green approach to the synthesis of high-quality graphene oxide flakes via electrochemical exfoliation of pencil core. *RSC Adv.* **3**, (2013).

5 Pei, S., Wei, Q., Huang, K., Cheng, H.-M. & Ren, W. Green synthesis of graphene oxide by seconds timescale water electrolytic oxidation. *Nat. Commun.* **9**, 145, (2018).

6 Parvez, K., Rincón, R. A., Weber, N.-E., Cha, K. C. & Venkataraman, S. S. One-step electrochemical synthesis of nitrogen and sulfur co-doped, high-quality graphene oxide. *Chem. Commun.* **52**, 5714-5717, (2016).

7 Wei, Q. *et al.* High Yield Controlled Synthesis of Nano-Graphene Oxide by Water Electrolytic Oxidation of Glassy Carbon for Metal-Free Catalysis. *ACS Nano* **13**, 9482-9490, (2019).

8 Tian, Z. *et al.* Facile electrochemical approach for the production of graphite oxide with tunable chemistry. *Carbon* **112**, 185-191, (2017).

9 Campéon, B. D. L. *et al.* Non-destructive, uniform, and scalable electrochemical functionalization and exfoliation of graphite. *Carbon* **158**, 356-363, (2020).

10 Sahoo, S. K. & Mallik, A. Synthesis and characterization of conductive few layered graphene nanosheets using an anionic electrochemical intercalation and exfoliation technique. *J. Mater. Chem. C* **3**, 10870-10878, (2015).

11 Cao, J. *et al.* Two-Step Electrochemical Intercalation and Oxidation of Graphite for the Mass Production of Graphene Oxide. *J. Am. Chem. Soc.* **139**,

17446-17456, (2017).

12 Chen, H., Du, W., Liu, J., Qu, L. & Li, C. Efficient room-temperature production of high-quality graphene by introducing removable oxygen functional groups to the precursor. *Chem. Sci.* **10**, 1244-1253, (2019).

13 Dong, L. *et al.* Reactivity-Controlled Preparation of Ultralarge Graphene Oxide by Chemical Expansion of Graphite. *Chem. Mater.* **29**, 564-572, (2017).

14 Yuan, H. *et al.* Highly Efficient Preparation of Graphite Oxide without Water Enhanced Oxidation. *Chem. Mater.* **33**, 1731-1739, (2021).

15 Marcano, D. C. *et al.* Improved Synthesis of Graphene Oxide. *ACS Nano* **4**, 4806-4814, (2010).

16 Hummers, W. S. & Offeman, R. E. Preparation of Graphitic Oxide. *J. Am. Chem. Soc.* **80**, 1339-1339, (1958).

17 Chen, J. *et al.* Synthesis of graphene oxide sheets with controlled sizes from sieved graphite flakes. *Carbon* **110**, 34-40, (2016).

18 Chen, J., Li, Y., Huang, L., Li, C. & Shi, G. High-yield preparation of graphene oxide from small graphite flakes via an improved Hummers method with a simple purification process. *Carbon* **81**, 826-834, (2015).

19 Chen, J., Yao, B., Li, C. & Shi, G. An improved Hummers method for eco-friendly synthesis of graphene oxide. *Carbon* **64**, 225-229, (2013).

20 Qian, X. *et al.* CdPS3 nanosheets-based membrane with high proton conductivity enabled by Cd vacancies. *Science* **370**, 596-600, (2020).

21 Zhou, J. *et al.* Layered Intercalation Materials. *Adv. Mater.* **33**, 2004557, (2021).

22 Leung, S. Y., Dresselhaus, M. S. & Dresselhaus, G. Infrared and Raman spectroscopy of graphite intercalation compounds. *Physica B+C* **105**, 375-380, (1981).

23 Dresselhaus, M. S. & Dresselhaus, G. Intercalation compounds of graphite. *Adv. Phys.* **51**, 1-186, (2002).

24 Beck, F., Jiang, J. & Krohn, H. Potential oscillations during galvanostatic

overoxidation of graphite in aqueous sulphuric acids. *J. Electroanal. Chem.* **389**, 161-165, (1995).

25 Bottomley, M. J., Parry, G. S., Ubbelohde, A. R. & Young, D. A. 1083. Electrochemical preparation of salts from well-oriented graphite. *J. Chem. Soc.*, 5674-5680, (1963).

26 Hennig, G. The Properties of the Interstitial Compounds of Graphite. I. The Electronic Structure of Graphite Bisulfate. *J. Chem. Phys.* **19**, 922-929, (1951).

27 Dimiev, A. M. & Tour, J. M. Mechanism of Graphene Oxide Formation. *ACS Nano* **8**, 3060-3068, (2014).

28 Inagaki, M., Iwashita, N. & Kouno, E. Potential change with intercalation of sulfuric acid into graphite by chemical oxidation. *Carbon* **28**, 49-55, (1990).

29 Khrapach, I. *et al.* Novel Highly Conductive and Transparent Graphene-Based Conductors. *Adv. Mater.* **24**, 2844-2849, (2012).

30 Dimiev, A. M., Bachilo, S. M., Saito, R. & Tour, J. M. Reversible Formation of Ammonium Persulfate/Sulfuric Acid Graphite Intercalation Compounds and Their Peculiar Raman Spectra. *ACS Nano* **6**, 7842-7849, (2012).

31 Ferrari, A. C. & Robertson, J. Interpretation of Raman spectra of disordered and amorphous carbon. *Phys. Rev. B* **61**, 14095-14107, (2000).

32 Zhao, W., Tan, P. H., Liu, J. & Ferrari, A. C. Intercalation of Few-Layer Graphite Flakes with FeCl3: Raman Determination of Fermi Level, Layer by Layer Decoupling, and Stability. *J. Am. Chem. Soc.* **133**, 5941-5946, (2011).

33 Huang, Y. *et al.* Preparation of spherical ultrafine zirconia powder in microemulsion system and its dispersibility. *Ceram. Int.* **30**, 675-681, (2004).

34 Junkin, M., Watson, J., Geest, J. P. V. & Wong, P. K. Template-Guided Self-Assembly of Colloidal Quantum Dots Using Plasma Lithography. *Adv. Mater.* **21**, 1247-1251, (2009).

35 Dimiev, A., Kosynkin, D. V., Alemany, L. B., Chaguine, P. & Tour, J. M. Pristine Graphite Oxide. *J. Am. Chem. Soc.* **134**, 2815-2822, (2012).

36 Kumar, P. V. *et al.* Scalable enhancement of graphene oxide properties by thermally driven phase transformation. *Nat. Chem.* **6**, 151-158, (2014).

37 Munuera, J. M. *et al.* Electrochemical Exfoliation of Graphite in Aqueous Sodium Halide Electrolytes toward Low Oxygen Content Graphene for Energy and Environmental Applications. *ACS Appl. Mater. Interfaces* **9**, 24085-24099, (2017).

38 Munuera, J. M. *et al.* High quality, low-oxidized graphene via anodic exfoliation with table salt as an efficient oxidation-preventing co-electrolyte for water/oil remediation and capacitive energy storage applications. *Appl. Mater. Today* **11**, 246-254, (2018).

39 Parveen, N., Ansari, M. O. & Cho, M. H. Simple route for gram synthesis of less defective few layered graphene and its electrochemical performance. *RSC Adv.* **5**, 44920-44927, (2015).

40 Jung, S. M., Mafra, D. L., Lin, C.-T., Jung, H. Y. & Kong, J. Controlled porous structures of graphene aerogels and their effect on supercapacitor performance. *Nanoscale* **7**, 4386-4393, (2015).

41 Taheri Najafabadi, A. & Gyenge, E. Synergistic production of graphene microsheets by simultaneous anodic and cathodic electro-exfoliation of graphitic electrodes in aprotic ionic liquids. *Carbon* **84**, 449-459, (2015).

42 Huang, X. *et al.* Low defect concentration few-layer graphene using a two-step electrochemical exfoliation. *Nanotechnology* **26**, 105602, (2015).

43 Marković, Z. M. *et al.* Semi-transparent, conductive thin films of electrochemical exfoliated graphene. *RSC Adv.* **6**, 39275-39283, (2016).

44 Chen, D. *et al.* Photosynergetic Electrochemical Synthesis of Graphene Oxide. *J. Am. Chem. Soc.* **142**, 6516-6520, (2020).

45 Liu, B. *et al.* Electrochemically Exfoliated Chlorine-doped Graphene for Flexible All-Solid-State Micro-Supercapacitors with High Volumetric Energy Density. *Adv. Mater.* **34**, 2106309, (2022).

46 Luo, Z., Lu, Y., Somers, L. A. & Johnson, A. T. C. High Yield Preparation of

Macroscopic Graphene Oxide Membranes. *J. Am. Chem. Soc.* **131**, 898-899, (2009).

47 Zhang, P., Zhou, J., He, P., Yang, S. & Ding, G. A one-pot strategy for highly efficient preparation of ultra-large graphene oxide. *Carbon* **191**, 477-485, (2022).

48 Xiaoliang, Z. *et al.* Risk assessment on chemical plants by the method of safety checklist analysis. *Process Saf. Prog.* **36**, 95-101, (2017).

49 Zhang, Q. et al. Defects boost graphitization for highly conductive graphene films. *Natl. Sci. Rev.* **10**, nwad147, (2023).
