# Supplementary material for: Control of water for high-yield and low-cost sustainable electrochemical synthesis of uniform monolayer graphene oxide
Source: Nat Commun. 2025 Jan 16;16:727. doi: 10.1038/s41467-025-56121-4 (PMC11739590; doi:10.1038/s41467-025-56121-4)
Supplement: Supplementary file 2 — Description of Additional Supplementary Files [file 41467_2025_56121_MOESM2_ESM.pdf]

Description of Additional Supplementary Files

File Name: Supplementary Movie 1

Description: In-situ microscopic observation of the emulsion electrolysis process at the SA-GIC electrode. An emulsion with a concentration of approximately 10 wt.% was gradually added to the SA-GIC-I under an applied voltage of 10 V to observe the electrolytic process. As a result, yellow spots slowly emerged on the blue surface of the SA-GIC-I.

File Name: Supplementary Movie 2

Description: In-situ microscopic observation of the deintercalation process at the SA-GIC electrode. To observe the deintercalation process, 100 µl of electrolyte with a concentration of 30 wt.% was added to SA-GIC-I while applying a voltage of 1.0 V. Along the water diffusion direction, the blue surface of SA-GIC-I has been converted to cyan-white.

File Name: Supplementary Movie 3

Description: In-situ microscopic observation of the efficient oxidation process at the SA-GIC electrode. To examine the moderate contact state of the aqueous SA electrolyte, 200 µL of the electrolyte with a concentration of 20 wt.% was added to SA-GIC-I under an applied voltage of 3.0 V. In the direction of water diffusion, the blue surface of SA-GIC-I rapidly and uniformly transformed to yellow.

File Name: Supplementary Movie 4

Description: In-situ microscopic observation of the uneven oxidation process of excessive aqueous SA electrolyte at the SA-GIC electrode. To observe the excessive amount of aqueous SA electrolyte contact state, 400 µl of electrolyte with a concentration of 30 wt.% was added to SA-GIC-I under an applied voltage of 2.5 V. Observing along the direction of water diffusion, the originally blue surface of SA-GIC-I transitioned into yellow and cyan-white. This color change indicates that deintercalation occurred simultaneously with the oxidation process.

File Name: Supplementary Movie 5

Description: Video of SA-GIC-I oxidation process in LME equipment. In the experiment, the oxidation process of SA-GIC-I was observed under an applied voltage of 2.8 V, with a sulfuric acid (SA) concentration of 30 wt.%, a liquid film thickness of 8 mm, and a liquid film rise rate of 1.9 mm min^{-1}. Under these conditions, the blue SA-GIC-I was successfully and stably converted to yellow graphite oxide, without any noticeable release of oxygen.
